# Inferring Phylogenetic Networks with Maximum Pseudolikelihood under Incomplete Lineage Sorting

**DOI:** 10.1371/journal.pgen.1005896

**Published:** 2016-03-07

**Authors:** Claudia Solís-Lemus, Cécile Ané

**Affiliations:** 1 Department of Statistics, University of Wisconsin-Madison, Madison, Wisconsin, United States of America; 2 Department of Botany, University of Wisconsin-Madison, Madison, Wisconsin, United States of America; Harvard University, UNITED STATES

## Abstract

Phylogenetic networks are necessary to represent the tree of life expanded by edges to represent events such as horizontal gene transfers, hybridizations or gene flow. Not all species follow the paradigm of vertical inheritance of their genetic material. While a great deal of research has flourished into the inference of phylogenetic trees, statistical methods to infer phylogenetic networks are still limited and under development. The main disadvantage of existing methods is a lack of scalability. Here, we present a statistical method to infer phylogenetic networks from multi-locus genetic data in a pseudolikelihood framework. Our model accounts for incomplete lineage sorting through the coalescent model, and for horizontal inheritance of genes through reticulation nodes in the network. Computation of the pseudolikelihood is fast and simple, and it avoids the burdensome calculation of the full likelihood which can be intractable with many species. Moreover, estimation at the quartet-level has the added computational benefit that it is easily parallelizable. Simulation studies comparing our method to a full likelihood approach show that our pseudolikelihood approach is much faster without compromising accuracy. We applied our method to reconstruct the evolutionary relationships among swordtails and platyfishes (*Xiphophorus*: Poeciliidae), which is characterized by widespread hybridizations.

## Author Summary

Phylogenetic networks display the evolutionary history of groups of individuals (species or populations) including reticulation events such as hybridization, horizontal gene transfer or migration. Here, we present a likelihood method to learn networks from molecular sequences at multiple genes. Our model accounts for several biological processes: mutations, incomplete lineage sorting of alleles in ancestral populations, and reticulations in the network. The likelihood is decomposed into 4-taxon subsets to make the analyses scale to many species and many genes. Our work makes it possible to learn large phylogenetic networks from large data sets, with a statistical approach and a biologically relevant model.

## Introduction

Evolutionary relationships are typically visualized in a tree, which implicitly assumes vertical transfer of genetic material from ancestors to descendants. However, not all species follow this paradigm. If genes can be horizontally transferred between some organisms, a tree is not a good representation of their history. Such reticulate events include hybridization, horizontal gene transfer or migration with gene flow, and require methods to infer phylogenetic networks. While a great deal of research has flourished for the inference of phylogenetic trees from different types of data, methods to infer phylogenetic networks are still limited and under development.

There are mainly two kinds of phylogenetic networks: implicit and explicit. Implicit networks–also called split networks–describe the discrepancy in gene trees, or other sources of data, and methods are well developed to reconstruct these networks [1–4]. These methods tend to be fast. However, implicit networks lack biological interpretation as the internal nodes do not represent ancestral species. Explicit networks, on the other hand, represent explicit reticulation events and each node represents an ancestral species. Combinatorial methods to infer explicit networks (which we call phylogenetic networks here) are fast but ignore gene tree error and incomplete lineage sorting (ILS) as a possible source of gene tree discordance (e.g. [5]). Model-based methods are most accurate but can be computationally challenging. They calculate the likelihood of an observed gene tree given a species network taking into account both reticulation and ILS [6–8]. Their scope was expanded in [9] to search for the most likely phylogenetic network based on multi-locus data (see also [10] for a different likelihood framework, where sites instead of genes are treated as independent and ILS is ignored). The likelihood-based method in [9], implemented in PhyloNet, provides a solid theoretical framework to estimate the maximum likelihood phylogenetic network from a set of gene trees. It has several advantages: it incorporates uncertainty on the gene trees estimated from sequence data, accounts for a background level of gene tree discordance due to ILS, and controls the complexity of the network with a cross validation step. However, its likelihood computation is heavy and becomes intractable when increasing the number of taxa or the number of hybridizations, making this method practical for small scenarios of up to about 10 species and 4 hybridizations in the network.

Here, we provide a fast statistical method to estimate phylogenetic networks from multi-locus data. We first present the theory for the pseudolikelihood of a network. We do so by deriving the proportion of the genome that has each 4-taxon tree (quartet concordance factors) as expected under the coalescent model extended by hybridization events, and we prove the generic identifiability of the model. We then use the observed quartet concordance factors as inferred from the multi-locus data to estimate the species network. Our method SNaQ (Species Networks applying Quartets) is implemented in our open-source software package PhyloNetworks in Julia and publicly available at https://github.com/crsl4.

Like PhyloNet, our method can incorporate uncertainty in estimated gene trees and gene tree discordance due to ILS. Our pseudolikelihood has computational advantages. It is simpler and more scalable to many species, compared to the full likelihood. It also scales to a large number of loci because estimation of gene trees can be highly parallelized, then summarized by only 3 tree frequencies on each 4-taxon subsets used as input in the pseudolikelihood. In simulations, our method showed good performance and scaled to scenarios for which PhyloNet could not run. We also used SNaQ to infer the evolutionary relationships between *Xiphophorus* fishes, from 1,183 loci across 24 taxa. Our results were congruent with [11] and refined the placement of some hybridizations found in that study. The analyses here presented show that SNaQ can enable scientists to incorporate organisms to the “tree of life” in parts that are more net-like than tree-like, and thus, complete a broader picture of evolution.

## Models

### Phylogenetic networks

Intuitively, a phylogenetic network is a phylogenetic tree with added hybrid edges, causing some nodes to have two parents (but see [12]). Phylogenetic networks can describe various biological processes causing gene flow from one population to another such as hybridization, introgression, or horizontal gene transfer. Hybridization occurs when individuals from 2 genetically distinct populations interbreed, resulting in a new separate population. Introgression, or introgressive hybridization, is the integration of alleles from one population into another existing population, through hybridization and backcrossing. Genes are horizontally transferred when acquired by a population through a process other than reproduction, from a possibly distantly related population. Although these three processes are biologically different, we do not make the distinction when modeling them with a network. In other words, our model takes into account all three biological scenarios, but those scenarios are not distinguishable in the estimated phylogenetic network unless more biological information is provided.

Just like phylogenetic trees, networks can be rooted or unrooted. A *rooted phylogenetic network* on taxon set *X* is a connected directed acyclic graph with vertices *V* = {*r*} ∪ *V*_*L*_ ∪ *V*_*H*_ ∪ *V*_*T*_, edges *E* = *E*_*H*_ ∪ *E*_*T*_ and a bijective leaf-labeling function *f*: *V*_*L*_ → *X* with the following characteristics. The root *r* has indegree 0 and outdegree 2. Any leaf *v* ∈ *V*_*L*_ has indegree 1 and outdegree 0. Any tree node *v* ∈ *V*_*T*_ has indegree 1 and outdegree 2. Any hybrid node *v* ∈ *V*_*H*_ has indegree 2 and outdegree 1. A tree edge *e* ∈ *E*_*T*_ is an edge whose child is a tree node. A hybrid edge *e* ∈ *E*_*H*_ is an edge whose child is a hybrid node. Unrooted phylogenetic networks are typically obtained by suppressing the root node and the direction of all edges. We also consider *semi-directed* unrooted networks, where the root node is suppressed and we ignore the direction of all tree edges, but we maintain the direction of hybrid edges, thus keeping information on which nodes are hybrids. The placement of the root is then constrained, because the direction of the two hybrid edges to a given hybrid node inform the direction of time at this node: the third edge must be a tree edge directed away from the hybrid node and leading to all the hybrid’s descendants. Therefore the root cannot be placed on any descendant of any hybrid node, although it might be placed on some hybrid edges.

We further assume that the true network is of *level-1*[1], i.e. any given edge can be part of at most one cycle. This means that there is no overlap between any two cycles (but see the Discussion). Refer to [1] for other types of evolutionary networks. Throughout this work, we denote by

- *n* the number of taxa,
- *h* the number of hybridization events and
- *k*_*i*_ the number of nodes in the undirected cycle created by the *i*th hybrid node.

**Fig 1:**
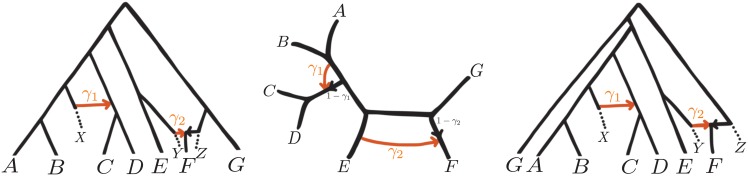
Example of rooted and semi-directed phylogenetic networks with *h* = 2 hybridization events and *n* = 7 sampled taxa. Inheritance probabilities *γ* represent the proportion of genes contributed by each parental population to a given hybrid node. Left: rooted network modelling several biological processes. Taxon F is a hybrid between two non-sampled taxa Y and Z with *γ*_2_ ≈ 0.50, and the lineage ancestral to taxa C and D has received genes introgressed from a non-sampled taxon X, for which *γ*_1_ ≈ 0.10. An alternative process at this event could be the horizontal transfer of only a handful of genes, corresponding to a very small fraction *γ*_1_ ≈ 0.001. Center: semi-directed network for the biological scenario just described. Although the root location is unknown, its position is constrained by the direction of hybrid edges (directed by arrows). For example, C, G or E cannot be outgroups. Right: rooted network obtained from the semi-directed network (center) by placing the root on the hybrid edge that leads to taxon F (labeled by 1 − *γ*_2_).

For example, in Fig 1 (center) *n* = 7, *h* = 2, *k*_1_ = 3 and *k*_2_ = 4. The main parameter of interest is the topology N of the semi-directed network. Like phylogenetic trees, this network can be rooted by a known outgroup. The other parameters of interest are **t**, the vector of branch lengths in coalescent units (see below), and a vector of inheritance probabilities *γ*, describing the proportion of genes inherited by a hybrid node from one of its hybrid parent (see Fig 1). Only identifiable branch lengths are considered in **t**. For example, with only one sequenced individual per taxon, the lengths of external edges are not identifiable and are not estimated.

#### Pseudolikelihood on a network

Pseudolikelihood has already been used to estimate phylogenetic trees under ILS [13], and here we extend the theory to networks. The pseudolikelihood of a network is based on the likelihood formulas of its 4-taxon subnetworks. These 4-taxon likelihoods are not independent but fast to compute. A quartet is a 4-taxon unrooted tree. For taxon set *s* = {*a*, *b*, *c*, *d*}, there are only three possible quartets, represented by the splits *q*_1_ = *ab*|*cd*, *q*_2_ = *ac*|*bd* and *q*_3_ = *ad*|*bc*.

The *concordance factor* (CF) of a given quartet (or split) is the proportion of genes whose true tree displays that quartet (or split) [14]. We use the term ‘CF’ as opposed to ‘probability’ to emphasize that CFs measure genomic support. Probabilities (such as posterior probabilities or bootstrap values) are most often thought to measure statistical uncertainty [15]. Intuitively, splits between natural evolutionary groups of organisms are recovered by most or all genes, and thus have high CFs. On the other hand, the presence of a hybrid would be captured by intermediate CFs. For example, if *a* is a hybrid intermediate between *b* and *c*, the CFs of *ab*|*cd* and *ac*|*bd* would be around 0.5 while the CF of *ad*|*bc* would be near 0.

The theoretical CFs (*CF*_*q*_1__, *CF*_*q*_2__, *CF*_*q*_3__) expected under the coalescent model are already known if the network is a species tree [16]. Interestingly, these CFs are independent of the root placement in the species tree, and are given by (1 − 2/3*e*^−*t*^, 1/3*e*^−*t*^, 1/3*e*^−*t*^) if the unrooted species tree is *q*_1_ = *ab*|*cd* with an internal edge of length *t* coalescent units. On a species network with reticulations, the probabilities of rooted gene trees was fully derived in [8] and more efficiently in [9]. But the probabilities of unrooted gene trees was not determined. We derive the quartet probabilities in the next section. In particular, they do not depend on the root placement in the network, which makes them simple and fast to compute.

To calculate the likelihood of a 4-taxon network from gene trees $G=\{G_{1},G_{2},...,G_{g}\}$ at *g* loci, we consider the number of gene trees **X** = (*X*_*q*_1__, *X*_*q*_2__, *X*_*q*_3__) that match each of the three quartets. Assuming unlinked loci, **X** follows a multinomial distribution with probabilities (*CF*_*q*_1__, *CF*_*q*_2__, *CF*_*q*_3__), the quartet CFs expected under the coalescent on the 4-taxon network. With a larger network on *n* ≥ 4 taxa, we consider all 4-taxon subsets *s* and combine the likelihood of each 4-taxon subnetworks to form the full network pseudolikelihood:

$$\begin{array}{c} L=\prod_{s\in S}{\left({\text{CF}}_{q_{1}}\right)}^{X_{q_{1}}}{\left({\text{CF}}_{q_{2}}\right)}^{X_{q_{2}}}{\left({\text{CF}}_{q_{3}}\right)}^{X_{q_{3}}} \end{array}$$ (1)

where S is the collection of all 4-taxon sets and *q*_*i*_ = *q*_*i*_(*s*) (*i* = 1, 2, 3) are the 3 quartet trees on *s*. In Eq (1), the data are summarized in the *X* values, and the candidate network governs the CF values, which we derive below.

#### Quartet CF for a 4-taxon network under ILS

For *h* = 1 hybridization, there are 5 different semi-directed 4-taxon networks (up to tip re-labelling). We describe here the expected quartet CFs (probabilities) for only one case and refer to S1 Text for the remaining cases.

**Fig 2:**
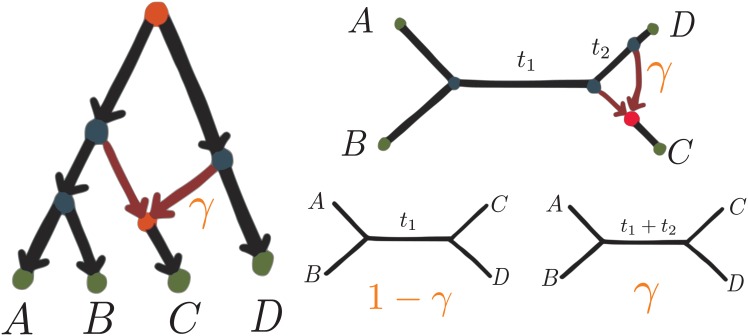
Rooted network (left) and its semi-directed version (top right). Quartet CFs expected under the network do not depend on the root placement, and are weighted averages of quartet CFs expected under the unrooted trees (bottom right).

Under the hybridization model described in [6, 8] and the network in Fig 2 (left), each gene from taxon *C* has probability *γ* of having descended from the hybridization edge sister to *D*, and probability 1 − *γ* of having descended from the original tree branch, sister to (*AB*). Therefore, the expected CFs are weighted averages of CFs obtained on 2 species tree with ILS. Because the quartet probabilities do not depend on the root placement in each species tree, they do not depend on the root placement in the original network either. Fig 2 (top right) shows the corresponding semi-directed network, and all rooted networks displaying it share the same quartet CFs, obtained from the coalescent models on the 2 unrooted species trees shown in Fig 2 (bottom right). These trees have the same topology but different branch lengths in this case. Therefore we get that ${\text{CF}}_{ab|cd}=\left(1-\gamma \right)\left(1-2/3\;e^{-t_{1}}\right)+\gamma \left(1-2/3\;e^{-t_{1}-t_{2}}\right)$ and the other 2 quartets occur with equal probabilities: ${\text{CF}}_{ac|bd}={\text{CF}}_{ad|bc}=\left(1-\gamma \right)\left(1/3\right)e^{-t_{1}}+\gamma \left(1/3\right)e^{-t_{1}-t_{2}}$.

**Fig 3:**
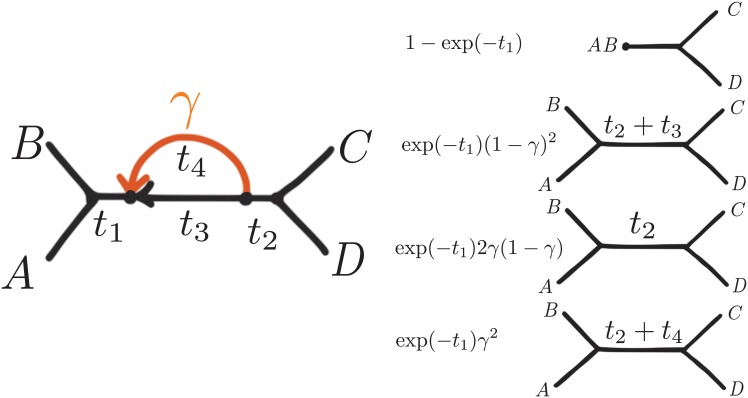
Example of a 4-taxon semi-directed network (left), with known direction of both hybrid edges but unspecified position of the root. The root can be placed on the internal edges with length *t*_2_, *t*_3_, *t*_4_, or on the external edges to C or D. The quartet CFs on this network are weighted averages of CFs under 4 trees with weights as shown (right).

On other semi-directed networks, more than 2 underlying unrooted species trees are needed if a hybrid node has more than one descendent taxon. In the network in Fig 3 for instance, the hybrid node has two descendants, *A* and *B*. Given this network, the computation of the CF for the major quartet *AB*|*CD* is as follows. First, *A* and *B* can coalesce along the branch of length *t*_1_ with probability $1-e^{-t_{1}}$. If they do not coalesce (with probability $e^{-t_{1}}$) then there are 3 options: both originated from the minor hybrid edge (probability *γ* each); both originated from the major hybrid edge (each with probability 1 − *γ*); or one (*A* or *B*) originated from the minor hybrid edge but the other (*B* or *A*) from the major. Assuming that each lineage’s origin is independent of the other, we get ${\text{CF}}_{AB|CD}=1-e^{-t_{1}}+e^{-t_{1}}({\left(1-\gamma \right)}^{2}\left(1-2/3e^{-t_{2}-t_{3}}\right)+2\gamma \left(1-\gamma \right)\left(1-2/3e^{-t_{2}}\right)+\gamma^{2}\left(1-2/3e^{-t_{4}-t_{2}}\right)).$ Therefore, this CF is a weighted average of CFs from the 4 species trees shown in Fig 3 (see S1 Text for all other cases).

With more than 1 hybridization (*h* > 1) there are an infinite number of semi-directed 4-taxon networks, but we can still calculate the quartet CFs if we assume that the cycles created by different reticulations do not share edges. We do so recursively on *h*, by reducing each network to an equivalent network with *h* = 0 or 1 and transformed branch lengths. For example, the network in Fig 2 leads to equal CFs of the 2 minor quartets *ac*|*bd* and *ad*|*bc*, so it is equivalent to the unrooted species tree *ab*|*cd* with internal branch length $t_{3}=-\log\left(\left(1-\gamma \right)e^{-t_{1}}+\gamma e^{-t_{1}-t_{2}}\right)$ to ensure $1/3\;e^{-t_{3}}={\text{CF}}_{ac|bd}$ given above. This new species tree and the original network have the same expected quartet CFs. The assumption of a level-1 network guarantees non-overlapping reticulation cycles, such that we can find an equivalent 4-taxon network with *h* = 0 or 1 and the same expected quartet CFs. We then just apply the network formulas above. The transformed branch lengths of the equivalent network are given in the S1 Text.

#### Detecting the presence of a hybridization

Identifiability is a basic requirement if one seeks to learn about parameters from data. Here our parameters are the network topology N, branch lengths *t* and inheritance values *γ*. We already know that quartet CFs do not depend on the root placement, so the rooted network is not identifiable and we only consider semi-directed networks N here. Our pseudolikelihood model would be identifiable if two different combinations of parameters (N,t,γ) and $\left(N^{\prime },t^{\prime },\gamma^{\prime }\right)$ yield different sets of quartet CFs. We show here and below that some reticulations and some parameters are impossible (or hard) to detect. This theory is used later to reduce the parameter space explored by our heuristic search, to avoid network and parameter combinations that are not identifiable.

**Fig 4:**
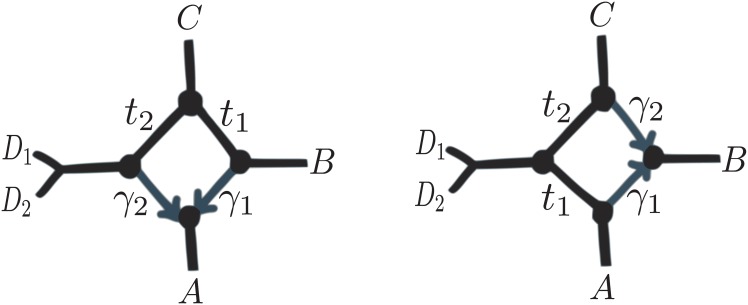
Networks with *k* = 4 nodes in the reticulation cycle and identical unrooted topologies. They differ in their hybrid position (left: good diamond, right: bad diamond I). If *D*_2_ is not sampled (*n* = 4), only $\gamma_{i}\left(1-e^{-t_{i}}\right)$ for *i* = 1, 2 are identifiable and the 2 networks are not distinguishable from each other.

On *n* = 4 taxa, we already showed that the network in Fig 2 is equivalent to a tree with some appropriate internal branch length. In fact, the same holds true for all 4-taxon networks with *k* = 2 or 3 nodes in their reticulation cycle: these reticulations cannot be detected. If *k* = 4 i.e. if the reticulation involves more distantly related taxa, then the presence of the hybridization can be detected based on the quartet CFs. However, networks with the same unrooted topology are unidentifiable from each other from only 4 taxa, like the 2 networks in Fig 4 if only *D*_1_ is sampled (*n* = 4). They only differ in the placement of the hybrid node, which is therefore not identifiable, even if the unrooted network and the presence of the reticulation is.

In general, for networks with *n* ≥ 4 taxa, we restrict our focus to the case when $N^{\prime }$ is the network topology obtained from N by removing a single hybrid edge of interest. The assumption of non-intersecting cycles allows us to study the detectability of this one hybrid edge given the other hybridizations in the network (see S1 Text). Assuming that all $\left(\begin{array}{c} n\\ 4 \end{array}\right)$ 4-taxon sets are used in the pseudolikelihood, the network N gives us $3\left(\begin{array}{c} n\\ 4 \end{array}\right)$ quartet CFs expected under the coalescent. The presence of the hybridization of interest can be detected if the quartet CFs from (N,t,γ) cannot all be equal to the quartet CFs from $\left(N^{\prime },t^{\prime },\gamma^{\prime }\right)$ simultaneously. We matched both systems from N and $N^{\prime }$ using Macaulay2 [17], and checked the values of (*t*, *γ*) and (*t*′, *γ*′) when the two systems of CFs were equal (see S1 Text for full details). Apart from the obvious case *γ* = 0 for the hybrid edge absent in $N^{\prime }$, we found that N and $N^{\prime }$ were not distinguishable when *t*_*b*_ = 0 or *t*_*b*_ = ∞ for some tree branches *b*, implying either a hard polytomy or a branch with no ILS and a reduction of the problem to a 4-taxon network. We can ignore these cases with the reasonable assumption

A1:t∈(0,∞)for all tree branches andγ∈(0,1).

**Fig 5:**
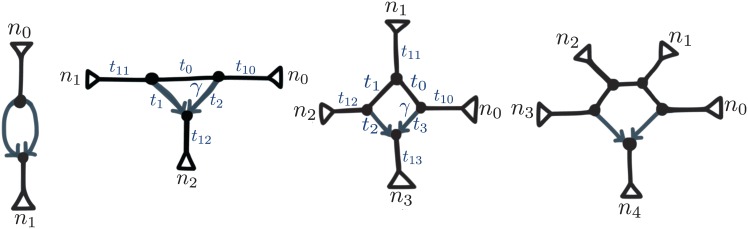
Networks with *k* nodes in a hybridization cycle: *k* = 2, 3, 4 and 5 from left to right. When *k* = 3, parameters are not identifiable. A good triangle corresponds to *n*_1_, *n*_2_, *n*_3_ ≥ 2, in which case setting *t*_12_ = 0 makes the other parameters identifiable. When *k* = 4, parameters are not all identifiable for the bad diamond I (*n*_0_ = *n*_2_ = *n*_3_ = 1 but *n*_1_ ≥ 2) and for the bad diamond II (*n*_0_ = *n*_1_ = *n*_2_ = 1 but *n*_3_ ≥ 2).

**A1** is not sufficient, however, to ensure that the presence of each hybridization in N can be detected. Increasing taxon sampling helps detect a hybridization only if the added taxa increase the size of the reticulation cycle. Namely, if the cycle only involves *k* = 2 nodes (see Fig 5), then N is not distinguishable from $N^{\prime }$, regardless of *n*. For *k* = 3, some hybridizations are detectable and some are not. If any two of the three subtrees defined by the hybridization cycle (Fig 5) have only one taxon, then the hybridization is not detectable. It is, if instead at least two subtrees contain more than one taxon. In general, hybridizations with *k* ≥ 4 can be detected if *n* ≥ 5. Here and below, we use the terms detectable or identifiable in their *generic* sense [18, 19], which simply means that some conditions on (*t*, *γ*) are required, like **A1**, but that all these conditions are met except on a subset of measure zero.

We further determined if the direction of a given hybrid edge was identifiable (in addition to its presence) when *n* = 5 and *k* = 4, in a case when the direction is not identifiable from 4 taxa. Fig 4 shows two networks that differ only in the placement of the hybrid node, but otherwise have the same unrooted topology. We proved that they yield different sets of quartet probabilities and therefore are distinguishable from each other, showing that the direction of the hybridization becomes identifiable when *n* ≥ 5.

#### Identifiability of branch lengths and heritabilities

We now turn to the case when $N^{\prime }=N$ to determine if *t* and *γ* are identifiable given a known network topology. Like before, we used Macaulay2 to determine under which conditions two different combinations of parameters (*t*, *γ*) and (*t*′, *γ*′) yield different sets of quartet probabilities for a fixed network N (see S1 Text for details).

Just as before, the identifiability depends on the type of network (Fig 5). With only 4 taxa, there are more parameters than equations (3 quartet CFs), so *t* and *γ* are not separately identifiable, so we focus first on the case with *n* ≥ 5.

If *n* ≥ 5, parameter identifiability is again easier if the reticulation involves more distantly related taxa. If *k* ≥ 5, all the parameters are identifiable. If *k* ≤ 3, parameters are not identifiable. If *k* = 4, parameters are identifiable if either *n*_0_ ≥ 2 (or *n*_2_, symmetrically), or if both *n*_1_ and *n*_3_ ≥ 2 (see Fig 5). We call this a *good diamond*. Parameters are not all identifiable in the remaining 2 cases, which we call *bad diamonds* I and II (see Fig 5). The bad diamond I already lacked identifiability under a different model in [20].

#### Practical consequences

A naive search for the most likely network would get stuck alternating between non-distinguishable networks or parameter sets. Hence we reduced the searchable space to only consider networks whose reticulations involve enough nodes. Indeed, all reticulations with *k* = 2 and most with *k* = 3 are either not detectable at all, or their parameters are not all identifiable. For hybridizations with *k* = 3, we only kept those with *n*_*i*_ ≥ 2 for all *i* = 0, 1, 2 (see Fig 5) and we enforced *t*_12_ = 0 to make the other 6 parameters identifiable. We denote this case as a *good triangle*. For bad diamonds I (*k* = 4), we reparametrized the 3 non-identifable values (*γ*, *t*_1_, *t*_0_) into 2 identifiable ones $\left(\gamma \left(1-e^{-t_{0}}\right),\left(1-\gamma \right)\left(1-e^{-t_{1}}\right)\right)$ (see S1 Text). For bad diamonds II, we set *t*_13_ = 0 and kept the other 5 parameters (*γ*, *t*_0_, *t*_1_, *t*_2_, *t*_3_).

### Network estimation procedure

The input for our method is a table of quartet CFs observed from multi-locus data (the *X* values in Eq (1)), across many or all 4-taxon subsets from the *n* taxa of interest.

#### Pseudolikelihood optimization

The maximum pseudolikelihood (MPL) estimate is the network, branch lengths *t* and *γ* heritabilities that maximize the pseudolikelihood Eq (1). This MPL optimization was fully implemented in SNaQ (Species Networks applying Quartets) and is part of our open source package PhyloNetworks in Julia [21]. The numerical optimization of branch lengths and *γ* parameters for a fixed topology is performed with a derivative-free methodology in the NLopt package for Julia. The heuristic optimization of the network topology uses a strategy similar to that in [9]. Given a fixed maximum number of hybridizations (*h*_*m*_), we search for the MPL network with at most *h*_*m*_ hybridizations. Since the pseudolikelihood can only improve when hybridizations are added, we expect the final network to have *h* = *h*_*m*_ exactly. A network is estimated for various values of *h*_*m*_, followed by a model selection procedure to select the appropriate number of hybridizations (see below). For a given *h*_*m*_, the search is initialized with a tree from a very fast quartet-based tree estimation method like ASTRAL [22] or Quartet MaxCut [23, 24]. The length of each branch is initialized using the average observed CF of the quartets that span that branch exactly, $\bar{\text{CF}}$, transformed to coalescent units by $t=-\log(1-3/2\;\bar{\text{CF}})$. The search then navigates the network space by altering the current network using one of 5 proposals, chosen at random: 1) move the origin of an existing hybrid edge, 2) move the target of an existing hybrid edge, 3) change the direction of an existing hybrid edge, 4) perform a nearest-neighbor interchange move (NNI) on a tree edge, and 5) add a hybridization if the current topology has *h* < *h*_*m*_. Any new proposed network is checked to verify that it is a semi-directed level-1 network with *h* ≤ *h*_*m*_ and with at least one valid placement for the root. More details on these moves are provided in S1 Text. Although the deletion of a current hybridrization is not proposed (because the MPL network should have *h* = *h*_*m*_), this deletion is still performed when suggested by the data, if the numerical optimization of parameters returns a $\hat{\gamma }=0$. In this case, the corresponding hybrid edge is removed and the search attempds to add it back at random in the neighborhood of the original hybrid edge. If this attempt fails for all neighbors, the hybridization is deleted entirely and the search continues from a network with 1 fewer hybridization. Similarly, if the numerical optimization returns a branch of length *t* = 0, an NNI move is proposed immediately on that branch. The search continues until the pseudolikelihood converges or until the number of consecutive failed proposals reaches a limit.

In [25], Huber et al. proved that the space of unrooted level-1 networks is connected by local subnetwork transfers, which generalize the NNI operations on trees and which are similar to our moves 1, 2 and 4. Although we do not have a formal proof that the MPL network can be reached from the starting tree using our proposals, the results in [25] suggest that it is the case.

#### Statistical uncertainty

There are two sources of uncertainty when we estimate CFs from sequence data. Gene trees are not observed directly but estimated, and only a finite number of genes can be sampled. Our preferred estimation of quartet CFs integrates over both sources of noise using BUCKy [26, 27], to estimate true gene tree conflict and discard conflict due to gene tree error. BUCKy returns estimated genome-wide CFs and their 95% credibility intervals. These CFs were shown to be most influenced by highly informative genes and least influenced by genes with large tree uncertainty [15]. Very briefly, MrBayes [28] is run on each gene separately and the full tree samples from all genes serve as input for BUCKy, which is run separately on each 4-taxon set. BUCKy has a prior probability of (1 + *α*/3)/(1 + *α*) that 2 genes share the same quartet tree. For example, choosing *α* = 1 amounts to assuming a prior concordance probability of 0.667, compared to 0.333 if gene trees matched just by chance.

A faster way to estimate CFs from sequences would be to use maximum likelihood with RAxML [29] (or maximum parsimony for faster estimation) on each gene separately, and then to simply count the number of genes that display each quartet tree. To account for tree uncertainty, one may drop any gene, for a given 4-taxon set, that does not have bootstrap support above some threshold (like 70%) for one of the 3 quartets. This method would not account for the uncertainty due to having a limited number of genes. With only 10 genes, for instance, the estimated CFs would necessarily be of low precision, of the form *i*/10 in the best case when all 10 gene trees are strongly supported.

If some genes are missing some taxa, the quartet CFs obtained on a given 4-taxon set can simply use the subset of genes that have sequences for the 4 taxa of interest. In most cases, a large number of genes can be included for each given 4-taxon sets, even if none of the genes have data across the full taxon set. Furthermore, the collection of 4-taxon sets with available CF data does not need to be exhaustive, as the sum in Eq (1) simply involves the sampled 4-taxon sets.

To measure uncertainty in the network, one may re-do the network analysis on bootstrap data sets. If we estimated credibility intervals for CFs with BUCKy, then 100 credible sets of quartet CFs can be obtained by sampling each CF from its posterior distribution, approximated by its credibility interval. If CFs were obtained using RAxML and observed quartet frequencies in gene trees, then bootstrap sets of quartet CFs could be obtained by sampling one bootstrap tree from each gene. To summarize the networks estimated from these bootstrap sets, we first calculated the support for edges being in the major tree: the tree obtained by suppressing the minor hybrid edge (with *γ* < 0.5) at each reticulation. We then summarized the support for the placement of each minor hybrid edge on that tree, considering 2 edges as equivalent if they are of the same type (hybrid or tree edges) and define the same clusters [9].

Uncertainty in the number of hybridizations *h* is more difficult to capture (see Discussion). We used here a slope heuristic to find where the network score changes from a sharp to a slow linear decrease as *h* increases. We also looked to see if the bootstrap support for successive reticulations dropped at the same *h* value.

## Results

### Simulated data

We carried out simulations to compare the speed and accuracy of SNaQ and PhyloNet. Given that PhyloNet uses the rooted and full gene trees, SNaQ can only be expected to perform as accurately as PhyloNet at best. Our simulations show that a pseudolikelihood approach does not compromise too much accuracy, but greatly improves speed.

We simulated *g* gene trees with ms [30] under four different networks: (*n*, *h*) = (6, 1), (6, 2), (10, 1) and (15, 3), with *γ* values set to 0.2 or 0.3 on each minor hybrid edge (see S1 Text) These network topologies were chosen at random by simulating a tree with *n* taxa under the coalescent, then choosing two edges at random for the origin and target of each hybridization and rejecting networks of level >1. On 6 taxa all reticulations were hard to reconstruct with *k* = 4, including a bad diamond I in the case *h* = 2. On 10 and 15 taxa, both networks also had a diamond, of the bad type II for *n* = 10. We varied the number of genes between 10 and 3000. All analyses were run on 2.7–3.5 GHz processors.

We first used the true simulated gene trees for inference. The rooted gene trees served as input for PhyloNet and the unrooted quartet CFs as observed in the *g* gene trees served as input for SNaQ. The semi-directed network returned by SNaQ was rooted by the outgroup species, when compatible with the estimated hybrid edges. Next, we used Seq-Gen [31] to simulate sequences of length 500 under HKY, *κ* = 2, A, C, G and T frequencies of 0.300414, 0.191363, 0.196748, 0.311475 and population mutation rate *θ* = 0.036, as in [9]. Gene trees were estimated with MrBayes [28] using 10^6^ generations sampled every 200, 25% burnin and an HKY model. The consensus trees (one per gene) served as input for PhyloNet. The posterior tree samples were then used in BUCKy [26, 27] for each 4-taxon set, to estimate quartet CFs and use them as input for SNaQ. For this pipeline, we used the tools implemened by [32] and available at https://github.com/nstenz/TICR. This procedure was replicated 30 times. The accuracy of each method was measured as the proportion of times that the estimated network matched the true network. To compare rooted networks we used the distance in [33], which is a metric on reduced networks (including level-1 networks) and is implemented in PhyloNet. We used it to detect equality between rooted networks, but not to measure how “close” networks were, because this distance is very sensitive to small differences such as a change in the direction of a hybrid edge.

**Fig 6:**
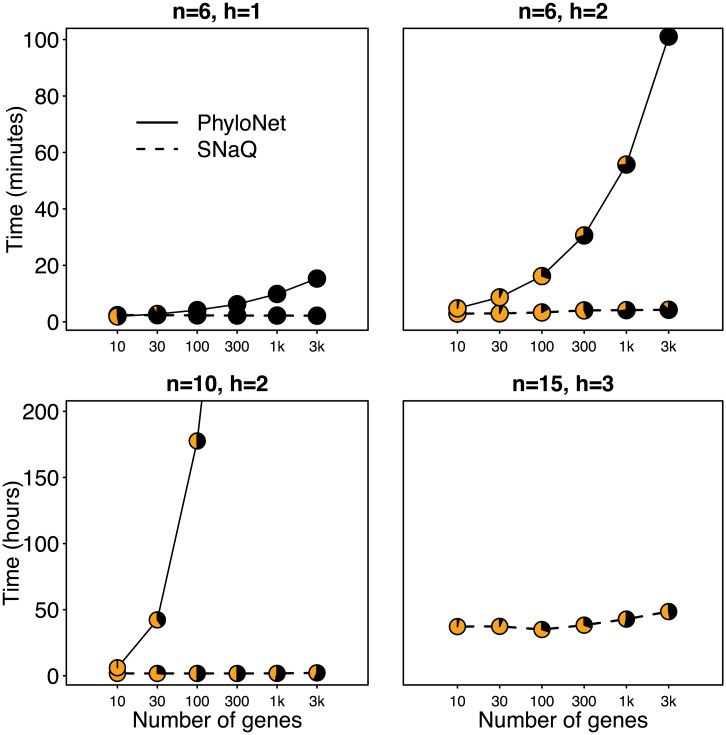
Performance (average computing time per replicate) of SNaQ and PhyloNet. in simulations using true gene trees on networks with *n* = 6, 10 or 15 taxa and *h* = 1, 2 or 3. Each replicate consisted of 10 independent runs with full optimization of branch lengths and inheritance probabilities for each run. Pie charts display accuracy (black: probability of recovering the true network). With *n* = 10 and 300 or more loci, or with *n* = 15, PhyloNet was too slow to run.

Fig 6 summarizes the accuracy and speed of SNaQ and PhyloNet. On 10 or 15 taxa PhyloNet was too slow to run (a single replicate with 10 taxa and 300 loci required over 400 hours), so we cannot provide a comparison of accuracy on these 2 larger networks.

**Fig 7:**
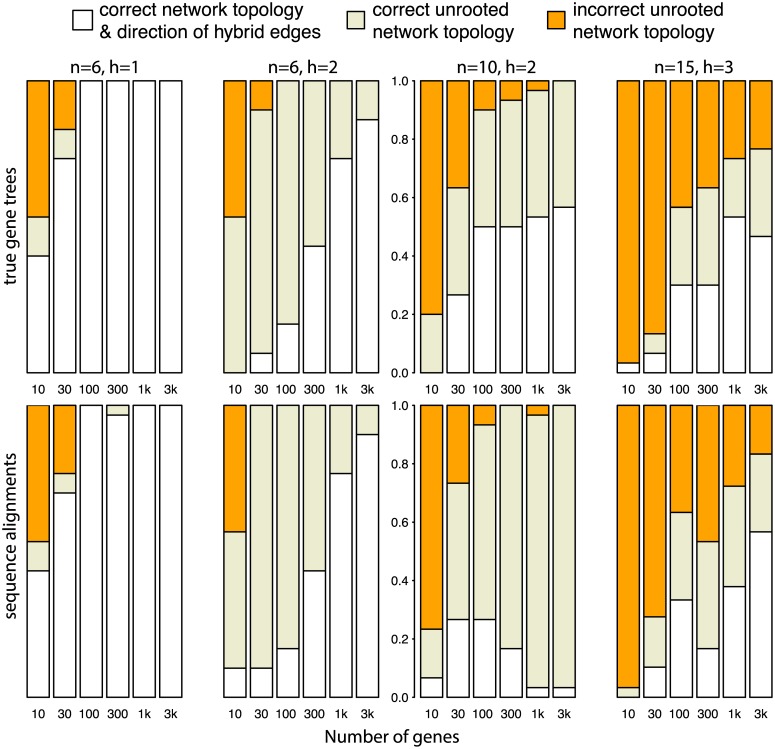
Accuracy of SNaQ in simulations using true gene trees or sequence alignments. Even when the semi-directed topology was not recovered, the unrooted topology was estimated correctly for most replicates using 30 loci or more and *h* ≤ 2.

For networks with *h* = 2 or more, the accuracy of SNaQ decreased. So, for each semi-directed network estimated by SNaQ, we determined if its unrooted topology matched that of the true network. Fig 7 shows that in the vast majority of cases when the directed network was incorrectly estimated, its unrooted topology was still correctly inferred from true gene trees and for *n* = 6 with estimated gene trees. For *n* ≥ 10, the inferred direction of hybrid edges degraded when gene trees were estimated. In most replicates on 10 taxa, this was because the bad diamond II near the root in the true network had a wrong estimated placement of the hybrid node.

**Fig 8:**
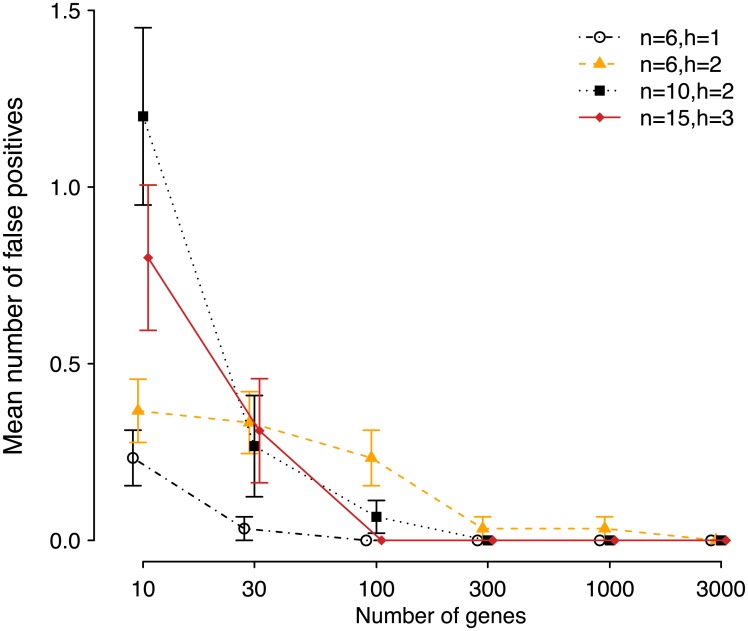
Accuracy of SNaQ to recover the major tree in the species network, from sequence alignments. The major tree is obtained by suppressing all minor hybrid edges (*γ* < 0.5) to capture the major vertical inheritance pattern. Accuracy is measured as half the Robinson-Foulds distance between the true and estimated tree, i.e. the number of incorrect edges in the estimated tree. A lot fewer genes are needed to accurately estimate the major vertical pattern, compared to the horizontal pattern.

To detemine which features in the network were correctly estimated, we extracted the major tree from each network, that is, the tree obtained by keeping the major hybrid edge and suppressing the minor hybrid edge at each hybrid node. We then compared the true major tree (from the true network) to the estimated major tree using the Robinson-Foulds distance (see Fig 8). The major tree was correctly estimated from 300 or more genes in all scenarios, except when *n* = 6, *h* = 2 and 300 genes (1 replicate out of 30) and 1000 genes (1 replicate out of 30). In both cases, the true major tree was displayed in the estimated network but the major hybrid edge was estimated as a minor edge with *γ* < 0.5. Therefore, the network’s “backbone”, i.e. the major vertical inheritance pattern, can still be estimated accurately even when the full network and hybrid edges are not (Fig 7).

**Fig 9:**
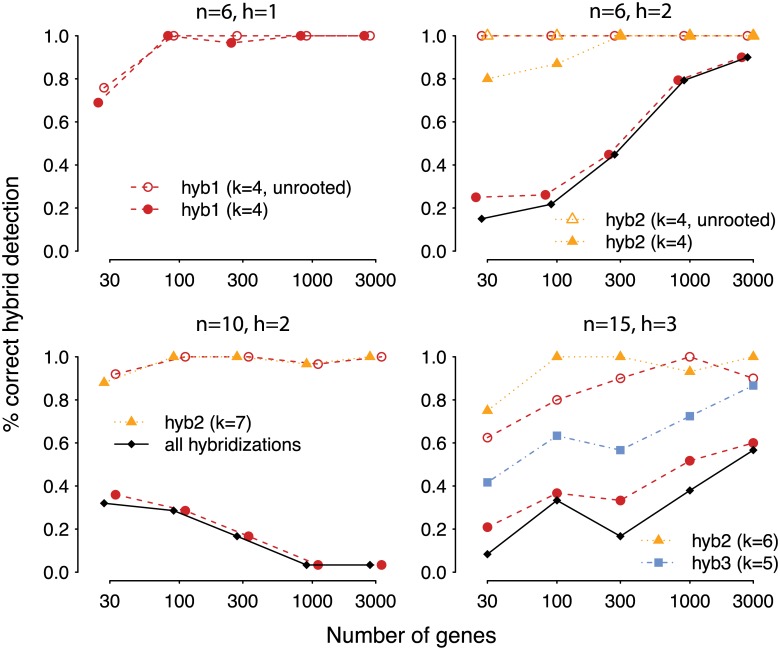
Accuracy of SNaQ to recover each hybridization. proportion of times each reticulation was correctly inferred (connecting the correct donor edge to the correct recipient edge in the major tree), among sequence alignments in which the major tree was recovered. Minor hybrid edges are numbered and colored as in S1 Text. For reticulations creating a cycle of *k* = 4 nodes, we also calculated the proportion of times that this undirected (or “unrooted”) cycle was correctly inferred, even though the identity and direction of hybrid edges in this cycle might be incorrect (empty symbols). The proportion of times that all hybridizations were correctly inferred (black lines) was low when a single hybridization with *k* = 4 was hard to recover (bad diamond I in case *n* = 6, *h* = 2, and bad diamond II in case *n* = 10).

Among cases when the major tree was correctly estimated, we determined the detection accuracy of each true hybridization event. To do so, we compared each estimated hybridization with the true hybridization of interest. In each network (true and estimated), we removed the other hybridizations by suppressing their minor hybrid edges and used the known outgroup to root both networks. We then calculated the hardwired cluster distance between the two resulting networks to determine if the estimated hybridization event matched the true hybridization of interest: connecting the same donor edge to the same recipient edge in the major tree (Fig 9). For *n* = 6, the hybridizations forming a good diamond were recovered with high accuracy from 100 genes, but the hybridization forming a bad diamond I (case *h* = 2) was very hard to recover, needing more than 1000 genes for an accurate inference of the hybrid edges’ direction. Still, the unrooted cycle was correctly estimated from 100 genes or more. For *n* = 10 and *n* = 15 taxa, the hybridization creating a cycle of *k* = 4 nodes was also very hard to detect with its correct direction, although its undirected cycle was accurately recovered from a few hundred genes. Hybridizations were recovered more accurately as their cycles spanned more nodes, with a high recovery rate for the hybridizations with *k* = 6 and *k* = 7 from 100 genes or more.

### *Xiphophorus* fishes evolution

**Fig 10:**
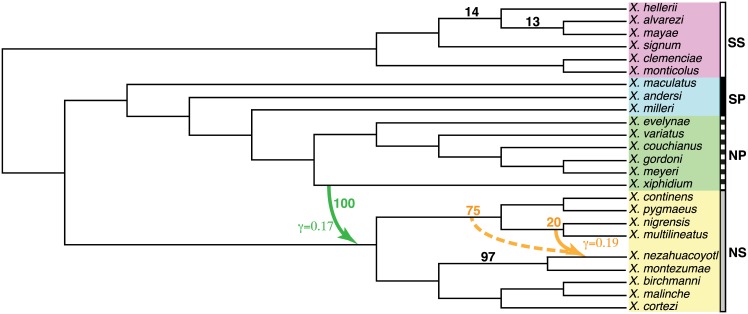
*Xiphophorus* reticulate evolution estimated with SNaQ. from 1183 genes, *h* = 2, rooted with the southern swordtails outgroup clade (SS). NS: northern swordtails, SP: southern platyfishes, NP: northern platyfishes. Black edges: major tree (including hybrid edges with *γ* > 0.5). Colored solid arrows: minor hybrid edges, annotated by their estimated *γ*. Black numbers: bootstrap support for edges in the major tree, if different from 100%. Colored numbers: bootstrap support for the placement of minor hybrid edges. One reticulation had 75% support for a different donor lineage (dotted arrow) than inferred from the original data.

We re-analyzed transcriptome data from [11] to reconstruct the evolutionary history of 24 swordtails and platyfishes (*Xiphophorus*: Poeciliidae). Based on high CFs of splits in conflict with their species tree followed by a series of ABBA-BABA tests [35], [11] concluded that hybridization or gene flow was widespread in the history of these tropical fishes. We re-analyzed their first set of 1183 transcripts. BUCKy was performed on each of the 10,626 4-taxon sets. The resulting quartet CFs were used in SNaQ, using *h*_*m*_ = 0 to 5 and 10 runs each. The network with *h* = 0 and the major tree in the network with *h* = 1 were identical to the total evidence tree in [11], with *X. xiphidium* placed within the grade of southern platyfishes (SP), making the northern platyfishes (NP) paraphyletic (see S1 Text). With *h* ≥ 2 the major tree was almost identical but with NP monophyletic (Fig 10) because *X. xiphidium* was found sister to the rest of the NP species, but involved in a reticulation (see below). With *h* ≥ 3, a reticulation within the southern swordtails (SS) was found consistently (*γ* = 0.43), but with a direction in conflict with SS being an outgroup clade. Its cycle had only *k* = 5 nodes, 4 of them leading to a single taxon (see S1 Text) so we suspect an error in the inferred hybrid node and gene flow direction. The extra 2 reticulations found with *h* = 4 and 5 had low *γ* values (in [0.006–0.16]).

The network scores (negative log-pseudolikelihood) decreased sharply from *h* = 0 to *h* = 2 then slightly and somewhat linearly (see S1 Text), suggesting that *h* = 2 best fits the fish data using a slope heuristic [45, 46]. The network estimated with *h* = 2 (Fig 10) found *X. xiphidium* involved in an ancient reticulation, contributing a proportion *γ* = 0.17 of genes to the lineage ancestral to northern swordtails (NS). This reticulation might explain the placement of *X. xiphidium* closer to the root in [11], from tree-based methods that do not account for potential gene flow. The second hybridization (*γ* = 0.20) was found from the population ancestral to *X. multilineatus* and *X. nigrensis* into *X. nezahuacoyotl*, and relates to a high CF found by [11] for a clade uniting *X. nezahuacoyotl* and the *nigrensis* group.

Bootstrap data sets were simulated by sampling each quartet CF from a uniform distribution on its 95% credibility interval (conservatively) then normalizing the sampled CFs across the 3 quartets on each 4-taxon set. For each bootstrap data set we estimated a network using 3 runs, and *h* = 3 (instead of 2) because the third inferred reticulation had a high *γ* (see S1 Text) and to assess the ability of the bootstrap procedure to identify the best *h* value. If the bootstrap was consistent with the slope heuristic, we expected high bootstrap support for the placement of the first 2 reticulations and lower support for the third. As expected, this third reticulation and network topology within the SS clade was variable among bootstrap networks (see S1 Text), suggesting uncertainty in the major tree within this clade (Fig 10). The rest of the tree was highly supported, as was the placement of the reticulation involving *X. xiphidium*. The reticulation involving *X. nezahuacoyotl* had split support for its donor lineage, with 75% support for a more ancestral lineage (Fig 10).

## Discussion

Many methods are being developped to understand organisms whose evolution behaves more net-like rather than tree-like. There is evidence of reticulation at all levels in the tree of life: deep among early prokaryotic and eukaryotic groups, to shallow among recently diverged species (e.g. [36–38]) or even among populations of the same species. Our new and fast statistical method to infer phylogenetic networks from multi-locus data could be used at these various levels in the tree of life.

### Network model and assumptions

Network inference is theoretically and computationally challenging. Split networks can be estimated rapidly, yet lack an evolutionary model and biological interpretability. [39] proposed a very fast distance-based approach to reconstruct topological ancestral recombination graphs (tARGs) from a long alignment, but the biological interpretability of tARGs is still limited. The evolution model in [8] uses an explicit network and satisfyingly accounts for various processes: reticulation events, deep coalescences, and substitutions. Yet a full likelihood estimation of large network (as in [9]) seems beyond computational reach. Our pseudolikelihood method offers an alternative, allowing the estimation of bigger and more complex networks while maintaining biological interpretability and a flexible evolutionary model.

We assumed a level-1 network throughout, where each hybrid node is part of a single cycle. This assumption is quite restrictive, but [40] showed that sequence data and gene trees on present-day species do not contain enough information to reconstruct complex networks, even from many loci. Therefore, some assumption has to be made to limit the network complexity. Extending our method to networks with intersecting cycles will need further work to restrict the search to candidate networks that are distinguishable from each other. Indeed, [40] show that different level-2 networks can have the exact same likelihood, and hence pseudolikelihood. So no method based on gene trees can ever decide which of these level-2 networks is true. Under a model without ILS, using full gene trees and branch length in substitutions per sites comparable across genes, [40] showed that level-1 networks are distinguishable but level-2 networks are not necessarily. Extending our approach to higher level networks, with or without ILS, will require extensive theory to work around this lack of identifiability.

Our approach allows for multiple individuals per species. All alleles from the same species simply need to be treated as a known and fixed polytomy in the network. Future work could include this and other topology constraints on the network, to reduce the computational burden when there are known phylogenetic relationships.

### Branch lengths

We allow hybrid edge lengths to be 0, but we do not constrain them to be 0 (unlike in [6, 8]) even though each gene flow event has to occur between contemporary populations. If one parental population went extinct or has no sampled descendants, the hybrid edge from this parent has a positive length in the observable network. A second reason is that a long branch can fit a population bottleneck, as might be expected in the formation of a new hybrid species. Not constraining hybrid branch lengths to 0 has a computational burden, however. Future implementations might enforce this constraint, when taxon sampling is thorough and extinction of parental populations can be ruled out.

By considering quartet topologies only, we ignored branch lengths in gene trees. This choice frees us from various assumptions. Using gene tree branch lengths, which are in substitutions per site, would require some assumption on gene rates to make branch lengths comparable across trees, and a molecular clock on gene trees. Other assumptions would also be needed on population sizes, shared or not across lineages. The recent approach in [41] should scale well to many taxa, but makes these strong assumptions because it requires accurate distances obtained from branch lengths in gene trees. On the contrary, our approach should be robust to rate variation across genes and across lineages, and does not require any assumption on population sizes.

### Identifiability of the topology

Yu et al. [8] already noted a lack of identifiability from rooted gene trees for reticulations with *k* = 3 from only 4 taxa (including the outgroup). We found a similar lack of identifiability from unrooted quartets if *n* < 5. In practice, some reticulations are hard to detect even with 5 or more taxa, if some branches are long with no ILS (close to violating **A1**). However, in these cases the unrooted topology of the network can still be recovered, even if the direction of gene flow and the placement of the hybrid node is not. Therefore, heuristic strategies that keep the unrooted network unchanged, or that just slightly modify it, may improve the search for the best network.

More tools are needed to study unrooted and semi-directed phylogenetic networks. For instance, no distance measure has been developed for such networks, that we know of. Distances between rooted networks would also be needed, that would be less sensitive to small changes in the unrooted or semi-directed topologies than the distance proposed in [33]. New notions of edge equivalence would also be needed on unrooted and semi-directed networks. It would help summarize a bootstrap sample of networks for instance, with no need for an outgroup.

We propose here a tree-based but informative summary by extracting the major tree from each network, obtained by dropping any minor hybrid edge (with inheritance *γ* < 0.5). Because this tree summarizes the major vertical inheritance pattern at each node, it can be considered an estimate of the species tree. We found that recovering the underlying species tree can be much easier (requiring fewer genes) than recovering the horizontal signal. Even if the species tree is the main purpose of a study, [34] showed that species-tree methods can be inconsistent in recovering the vertical signal if there is gene flow, so using a network can be beneficial to avoid the possible inconsistency of tree-based coalescent methods.

### Missing data

All data analyzed here had full taxon sampling from each gene, and we were able to use all 4-taxon sets. Future work could assess the impact of missing data (gene sequences, or 4-taxon sets) on the method’s accuracy. Missing 4-taxon sets will be necessary for large networks, because the number of 4-taxon sets grows very rapidly with the number of taxa (∼*n*^4^/24). With many taxa, one may randomly select a collection of 4-taxon sets and/or choose them specifically. SNaQ calculates the number of quartets involving each taxon and provides information about under-represented taxa, if any. With many individuals per species, one may greatly reduce the collection of 4-taxon sets to be analyzed by randomly sampling from those containing at most one individual per species. If the assignment of individuals to species is correct, any 4-taxon set containing 2 individuals from the same species would be non-informative about the species-level relationships. This strategy is used in [42] to infer species trees under ILS.

### 0.1 Uncertainty in the number of hybridizations

Model selection is necessary to estimate the number of hybridizations *h*, because the pseudolikelihood is bound to improve as *h* increases, like the likelihood or parsimony score in [43]. We used here the log pseudolikelihood profile with *h*. A sharp improvement is expected until *h* reaches the best value and a slower, linear improvement thereafter. Such data-driven slope heuristics can indeed be used with contrast functions (like pseudolikelihoods) for model selection in regression frameworks [45, 46].

Information criteria have already been used to select *h* (e.g. [44]), but these criteria are inappropriate if the full likelihood is replaced by a pseudolikelihood. Theory is missing to compare the pseudolikelihoods of different networks, because of the possible correlation between quartets from different 4-taxon sets. It can be shown, however, that quartets from two 4-taxon sets *s*_1_ and *s*_2_ are independent if *s*_1_ and *s*_2_ overlap by at most one taxon and if the true 4-taxon subnetworks share no internal edges. Future work could exploit this partial independence to construct hypothesis tests.

Cross validation has been proposed by [9], and was shown to have good performance. In our framework, the cross-valication error could be measured from the difference between the quartet CFs observed in the validation subset and the quartet CFs expected from the network estimated on the training set. Because *K*-fold cross-validation requires partitioning the loci into *K* subsets and re-estimating a network *K* times at each *h* value, this approach can be computationally heavy.

Finally, [32] proposed a goodness-of-fit test, also based on quartet CFs, to determine if a tree with ILS fits the observed data or if a network is needed instead. This test could be extended to networks, to decide if a given *h* provides an adequate fit. One advantage to this approach is that testing the adequacy of a given *h* does not require to estimate a larger network with *h* + 1 hybridizations, whereas other approaches above would require estimation of both networks in order to decide that the simpler network is sufficient.

### Pseudolikelihood with rooted triples

After submission, we learned about similar work using subnetworks and a pseudolikelihood approach [47], which scales to many taxa. In [47], the pseudolikelihood is based on rooted triples whereas we use unrooted quartets. There are fewer triples, so the method in [47] is potentially faster. However, fewer triples means less information. For example, the networks *Ψ*_1_ and *Ψ*_2_ shown in Fig. 2 of [47], which are not distinguishable from triplets, are in fact distinguishable from quartets (see S1 Text). Our thorough study of the network identifiability allowed us to implement a search that avoids jumping between networks that are not distinguishable, which facilitates convergence. The downside of our approach is the assumption of a level-1 network. Instead, [47] do not assume any restriction on the network. Finally, our method does not require rooted gene trees as input, which we view as a major advantage because rooting errors are avoided.

## Supporting Information

S1 Text Supporting information file that contains formal definitions, proofs of network identifiability results, more details on the heuristic network search, on the simulation study and on the fish network analysis. Quartet CFs under the coalescent with hybridizationTopology identifiabilityParameter identifiabilityHeuristic search in the space of networksIdentifiability from quartets versus triplesSimulated data*Xiphophorus* fish network analysis
(PDF)
