# Supplementary material for: Inferring Phylogenetic Networks with Maximum Pseudolikelihood under Incomplete Lineage Sorting
Source: PLoS Genet. 2016 Mar 7;12(3):e1005896. doi: 10.1371/journal.pgen.1005896 (PMC4780787; doi:10.1371/journal.pgen.1005896)
Supplement: S1 Text — Quartet CFs under the coalescent with hybridizationTopology identifiabilityParameter identifiabilityHeuristic search in the space of networksIdentifiability from quartets versus triplesSimulated dataXiphophorus fish network analysis (PDF) [file pgen.1005896.s001.pdf]

Supporting Information: Inferring phylogenetic networks with maximum pseudolikelihood under incomplete lineage sorting

Claudia Solís-Lemus and Cécile Ané

Contents

1 Quartet CFs under the coalescent with hybridization **2**
1.1 Quartet CFs for a 4-taxon network with one hybridization 2
1.2 Subnetwork equivalence for quartet CFs 3
1.3 Four-taxon networks with more than one hybridization 3

2 Topology identifiability **4**
2.1 5-taxon network: topology identifiability 4
2.2 n-taxon network: topology identifiability 5

3 Parameter identifiability **7**
3.1 Identifiability of parameters in a 5-taxon network 7
3.2 n-taxon network: parameter identifiability when $h = 1$ 9
3.3 Parameter identifiability in level-1 networks 10

4 Heuristic search in the space of networks **11**

5 Identifiability from quartets versus triples **12**

6 Simulated data **15**

7 *Xiphophorus* fish network analysis **16**

1 Quartet CFs under the coalescent with hybridization

1.1 Quartet CFs for a 4-taxon network with one hybridization

A argument similar to what is described in the main text was applied to all 4-taxon networks with $h = 1$. The results are summarized below.

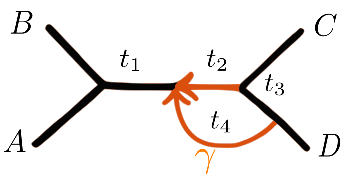

Network 1

$$
\begin{aligned}
CF_{AB|CD} &= (1-\gamma)^2(1-2/3\exp(-t_1-t_2)) \\
&+ 2\gamma(1-\gamma)(1-\exp(-t_1)+1/3\exp(-t_1-t_3)) \\
&+ \gamma^2(1-2/3\exp(-t_1-t_4)) \\
CF_{AC|BD} &= (1-\gamma)^2(1/3\exp(-t_1-t_2)) \\
&+ \gamma(1-\gamma)\exp(-t_1)(1-1/3\exp(-t_3)) \\
&+ \gamma^2(1/3\exp(-t_1-t_4)) \\
CF_{AD|BC} &= (1-\gamma)^2(1/3\exp(-t_1-t_2)) \\
&+ \gamma(1-\gamma)\exp(-t_1)(1-1/3\exp(-t_3)) \\
&+ \gamma^2(1/3\exp(-t_1-t_4))
\end{aligned}
$$

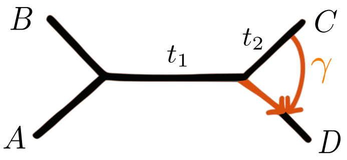

Network 2

$$
\begin{aligned}
CF_{AB|CD} &= (1-\gamma)(1-2/3\exp(-t_1)) + \gamma(1-2/3\exp(-t_1-t_2)) \\
CF_{AC|BD} &= (1-\gamma)1/3\exp(-t_1) + \gamma 1/3\exp(-t_1-t_2) \\
CF_{AD|BC} &= (1-\gamma)1/3\exp(-t_1) + \gamma 1/3\exp(-t_1-t_2)
\end{aligned}
$$

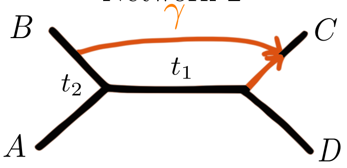

Network 3

$$
\begin{aligned}
CF_{AB|CD} &= (1-\gamma)(1-2/3\exp(-t_1)) + \gamma(1/3\exp(-t_2)) \\
CF_{AC|BD} &= (1-\gamma)1/3\exp(-t_1) + \gamma(1-2/3\exp(-t_2)) \\
CF_{AD|BC} &= (1-\gamma)1/3\exp(-t_1) + \gamma 1/3\exp(-t_2)
\end{aligned}
$$

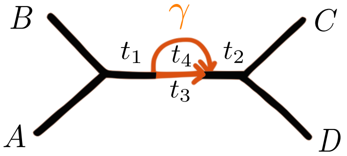

Network 4

$$
\begin{aligned}
CF_{AB|CD} &= (1-\gamma)^2(1-2/3\exp(-t_1-t_2-t_3)) \\
&+ 2\gamma(1-\gamma)(1-2/3\exp(-t_1-t_2)) \\
&+ \gamma^2(1-2/3\exp(-t_1-t_2-t_4)) \\
CF_{AC|BD} &= (1-\gamma)^2(1/3\exp(-t_1-t_2-t_3)) \\
&+ 2\gamma(1-\gamma)(1/3\exp(-t_1-t_2)) \\
&+ \gamma^2(1/3\exp(-t_1-t_2-t_4)) \\
CF_{AD|BC} &= (1-\gamma)^2(1/3\exp(-t_1-t_2-t_3)) \\
&+ 2\gamma(1-\gamma)(1/3\exp(-t_1-t_2)) \\
&+ \gamma^2(1/3\exp(-t_1-t_2-t_4))
\end{aligned}
$$

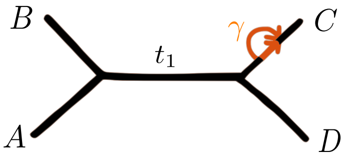

Network 5

$$
\begin{aligned}
CF_{AB|CD} &= 1-2/3\exp(-t_1) \\
CF_{AC|BD} &= 1/3\exp(-t_1) \\
CF_{AD|BC} &= 1/3\exp(-t_1)
\end{aligned}
$$

1.2 Subnetwork equivalence for quartet CFs

We show here how a level-1 four-taxon network with any number of hybridizations can be reduced to an equivalent network with $h = 0$ or 1 hybridizations only, where equivalence means that both networks give the same quartet CFs. The network is reduced by induction on h, by replacing each "blob" in the network (a set of nodes and edges along a given cycle [4]) by a simpler network. To simplify notations we use $z_i = 1 - \exp(-t_i)$. A subnetwork is of *type 1* if it leads to two equal minor CFs, and is therefore equivalent to a subnetwork with no hybridization. A subnetwork is of *type 2* if it leads to three different quartet CFs, and is not equivalent to a tree. We summarize below all the possible subnetwork configurations on 4 taxa (on the left in each figure) and the equivalent form (on the right), with the formula giving the branch length in the equivalent network to obtain the same CFs from both networks.

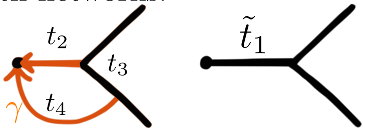

$$\exp(-\tilde{t}_1) := 1 + \gamma z_3 - \gamma^2 z_4 - \gamma^2 z_3 - (1-\gamma)^2 z_2$$

Subnetwork 1 (type 1)

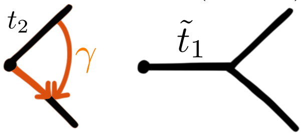

$$\exp(-\tilde{t}_1) := 1 - \gamma z_2$$

Subnetwork 2 (type 1)

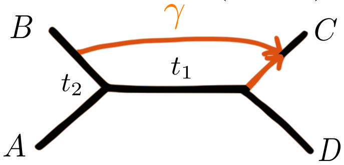

$$3CF_{AB|CD} = 1 - 2(1-\gamma)z_1 - \gamma z_2$$
$$3CF_{AC|BD} = 1 + 2\gamma z_2 - (1-\gamma)z_1$$
$$3CF_{AD|BC} = 1 - (1-\gamma)z_1 - \gamma z_2$$

Subnetwork 3 (type 2)

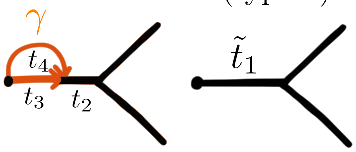

$$\exp(-\tilde{t}_1) := \exp(-t_2)[1 - \gamma^2 z_4 - (1-\gamma)^2 z_3]$$

Subnetwork 4 (type 1)

1.3 Four-taxon networks with more than one hybridization

We can use the subnetwork equivalence just described to account for multiple hybridizations on the same network (see example on Fig. S1 left). Even when none of the hybridizations are of type 1 on the full network, some hybridizations may become of type 1 after pruning taxa. Fig. S1 (right) shows such an example: when reduced to the four taxa 1, 2, 4 and 10, the network in Fig. S5 (d) has two hybridizations of type 1. Both can be removed with the subnetwork equivalence described in the previous section.

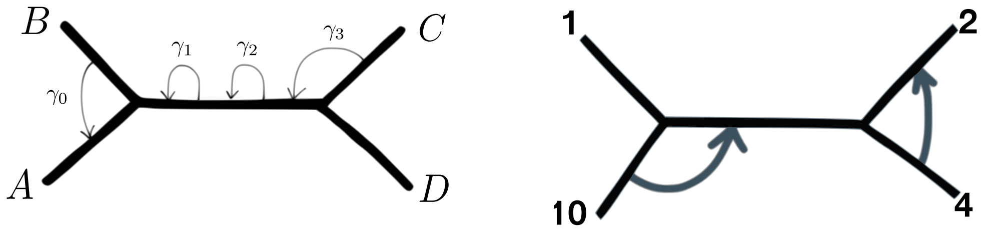

Figure S1: Left: Network on 4 taxa with multiple hybridizations of type 1. This network is equivalent to a 4-taxon tree (without hybridizations) in terms of expected CFs. Right: 4-taxon network extracted from the network in Fig. S5 (d) with 2 hybridizations of type 1. This network is equivalent to a 4-taxon tree (without hybridizations) in terms of expected CFs.

2 Topology identifiability

2.1 5-taxon network: topology identifiability

Assume first $h = 1$. A network with $n = 5$ taxa has $\binom{5}{4} = 5$ four-taxon subsets, and each one has three possible quartets. Each quartet CF expected on this network is given in previous section 1.1. Thus, a 5-taxon network defines $3 * 5 = 15$ quartet CF equations. The same is true for a 5-taxon species tree obtained by removing the hybridization event in the network, but with different CF formulas.

The hybridization in the network is identifiable if the same set of quartet CF values cannot solve simultaneously the system of equations for the network and the system of equations for the tree. If there exists a set of CF values that can solve both systems of equations, then we cannot identify the network from the tree based on quartet CFs. Thus, the conditions under which $CF_{network} = CF_{tree}$ have a solution allow us to identify the conditions under which the network is not identifiable from the tree. Take, for example, the 5-taxon network

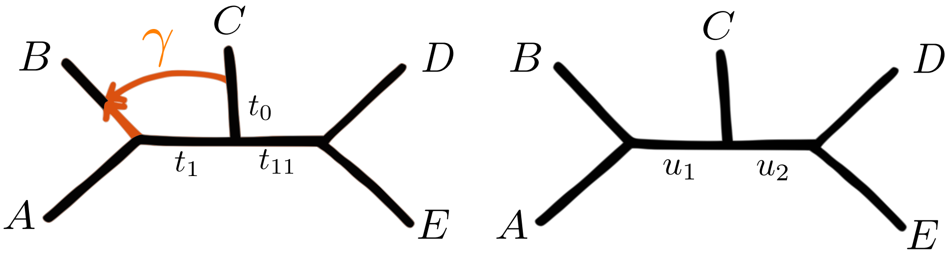

Figure S2: The network (left) is identifiable from the tree (right) based on quartet CFs, provided that $t_0 > 0$, $t_1 > 0$, $t_{11} < \infty$ and $\gamma \in (0, 1)$, for any value of u_1 and u_2.

in Fig. S2 (left) with system of CF equations in table S1. To prove that it is identifiable from the tree in Fig. S2 (right), we computed the 15 quartet CFs $c_1, c_2, ..., c_{15}$ for the tree with the formulas in table S2 for given values of branch lengths. Then, with aid of Macaulay2, we investigated if the set of equations for the network would have a solution when equal to

Table S1: System of concordance factors equations for 5-taxon network in Fig. S2 (left)

$ABCD$	$CF_{AB\|CD} = (1-\gamma)(1-2/3\exp(-t_1)) + \gamma(1/3\exp(-t_0))$ $CF_{AD\|BC} = (1-\gamma)(1/3\exp(-t_1)) + \gamma(1-2/3\exp(-t_0))$ $CF_{AC\|BD} = (1-\gamma)(1/3\exp(-t_1)) + \gamma(1/3)\exp(-t_0)$
$ABDE$	$CF_{AB\|CE} = (1-\gamma)(1-2/3\exp(-t_1)) + \gamma(1/3\exp(-t_0))$ $CF_{AE\|BC} = (1-\gamma)(1/3\exp(-t_1)) + \gamma(1-2/3\exp(-t_0))$ $CF_{AC\|BE} = (1-\gamma)(1/3\exp(-t_1)) + \gamma(1/3)\exp(-t_0)$
$ABDE$	$CF_{AC\|DE} = (1-\gamma)(1-2/3\exp(-t_1-t_{11})) + \gamma(1-2/3\exp(-t_{11}))$ $CF_{AD\|CE} = (1-\gamma)(1/3\exp(-t_1-t_{11})) + \gamma(1/3\exp(-t_{11}))$ $CF_{AE\|CD} = (1-\gamma)(1/3\exp(-t_1-t_{11})) + \gamma(1/3\exp(-t_{11}))$
$ACDE$	$CF_{AC\|DE} = 1-2/3\exp(-t_{11})$ $CF_{AD\|CE} = 1/3\exp(-t_{11})$ $CF_{AE\|CD} = 1/3\exp(-t_{11})$
$BCDE$	$CF_{AC\|DE} = (1-\gamma)(1-2/3\exp(-t_{11})) + \gamma(1-2/3\exp(-t_0-t_{11}))$ $CF_{AD\|CE} = (1-\gamma)(1/3\exp(-t_{11})) + \gamma(1/3\exp(-t_0-t_{11}))$ $CF_{AE\|CD} = (1-\gamma)(1/3\exp(-t_{11})) + \gamma(1/3\exp(-t_0-t_{11}))$

those CF values. It turns out that regardless of the values of the tree branch lengths u_1, u_2, the 5-taxon network is identifiable from the tree as long as $t_0 > 0$, $t_1 > 0$, $t_{11} < \infty$ and $\gamma \in (0, 1)$.

A similar study was done to every possible 5-taxon network with one hybridization to conclude that if $t_i > 0$ for all tree edge i and if $\gamma \in (0, 1)$, then the network is generically identifiable from the tree (as long as the number of nodes in the hybridization cycle is $k \geq 3$).

2.2 *n*-taxon network: topology identifiability

To generalize to $n \geq 5$ taxa, not all $\binom{n}{4} \times 3$ quartet CF equations are needed. In Fig. S3, the networks are ordered by the number of nodes in the hybridization cycle. Ignoring the case with $k = 2$ which is not identifiable from a tree, and assuming that the network is level-1, the hybridization cycle in the network is attached to k subnetworks, each represented by a small triangle with a given number of taxa n_i. With $k = 3$ nodes in the hybridization cycle for example, the network has three subgraphs, each with n_0, n_1, n_2 taxa respectively. If $n_0 \geq 3$ in the top-right subnetwork, one can form a 4-taxon subset by taking three taxa from this subnetwork and one taxon from any of the other 2 subnetworks. However, the three CF formulas for such a 4-taxon subset do not involve any of the parameters near the hybridization cycle of interest. That is, the CFs resulting from choosing three taxa from one subtree and one taxon from another subtree yield no information about the hybridization event of interest. In other words, we can ignore all the equations that involve three taxa from the same subgraph.

Thus, to study the identifiability of an n-taxon network, it suffices to consider only the subset of quartets involving $n_i \leq 2$ taxa for any subnetwork i. If only one tip is taken for

Table S2: System of concordance factors equations for 5-taxon unrooted tree in Fig. S2 (right)

$ABCD$	$CF_{AB\|CD} = 1 - 2/3\, e^{-u_1}$<br>$CF_{AD\|BC} = 1/3\, e^{-u_1}$<br>$CF_{AC\|BD} = 1/3\, e^{-u_1}$
$ABDE$	$CF_{AB\|CE} = 1 - 2/3\, e^{-u_1}$<br>$CF_{AE\|BC} = 1/3\, e^{-u_1}$<br>$CF_{AC\|BE} = 1/3\, e^{-u_1}$
$ACDE$	$CF_{AC\|DE} = 1 - 2/3\, e^{-u_2}$<br>$CF_{AD\|CE} = 1/3\, e^{-u_2}$<br>$CF_{AE\|CD} = 1/3\, e^{-u_2}$
$BCDE$	$CF_{BC\|DE} = 1 - 2/3\, e^{-u_2}$<br>$CF_{BD\|CE} = 1/3\, e^{-u_2}$<br>$CF_{BE\|CD} = 1/3\, e^{-u_2}$
$ABDE$	$CF_{AB\|DE} = 1 - 2/3\, e^{-u_1-u_2}$<br>$CF_{AD\|BE} = 1/3\, e^{-u_1-u_2}$<br>$CF_{AE\|BD} = 1/3\, e^{-u_1-u_2}$

subnetwork i, then hybridizations in this subnetwork do not affect the quartet CFs. If the 4-taxon set has $n_i = 2$ tips from subnetwork i, then there could be a hybridization involving them within this subnetwork. Because the network is of level 1, however, this hybridization has to be of type 1 (see Section 1.2) and can be removed without affecting the CFs, using a transformed branch length leading to subnetwork i. Thus, we can study the identifiability of a given hybridization of interest ignoring hybridizations in the subnetworks. Using Macaulay2, we found the same sufficient conditions as with $n = 5$ for generic identifiability of the topology: $t \in (0, \infty)$ for all tree branch lengths and $\gamma \in (0, 1)$. We give below weaker, but still sufficient conditions for each value of k. The n-taxon network with one hybridization and k nodes in this hybridization cycle is generically identifiable from a tree with the same topology but the hybridization removed if:

$$\boldsymbol{k=3}:\quad t_{10}, t_{11}, t_{12} < \infty;\;\; t_0 > 0;\;\; \gamma \in (0,1)$$
$$(1-\gamma)(1-\exp(-t_1)) \neq \gamma((1-\exp(-t_2)) + (1-\exp(-t_0)))$$
$$\gamma(1-\exp(-t_2)) \neq (1-\gamma)((1-\exp(-t_0)) + (1-\exp(-t_1)))$$

$$\boldsymbol{k=4}:\quad t_{12}, t_{13} < \infty;\;\; t_0, t_1 > 0;\;\; \gamma \in (0,1)$$
$$\gamma(1-\exp(-t_3)) \neq (1-\gamma)((1-\exp(-t_1)) + (1-\exp(-t_2)))$$

$$\boldsymbol{k=5}:\quad t_{13}, t_{14} < \infty;\;\; t_0, t_1, t_2 > 0;\;\; \gamma \in (0,1)$$
$$\gamma(1-\exp(-t_4)) \neq (1-\gamma)((1-\exp(-t_2)) + (1-\exp(-t_3)))$$

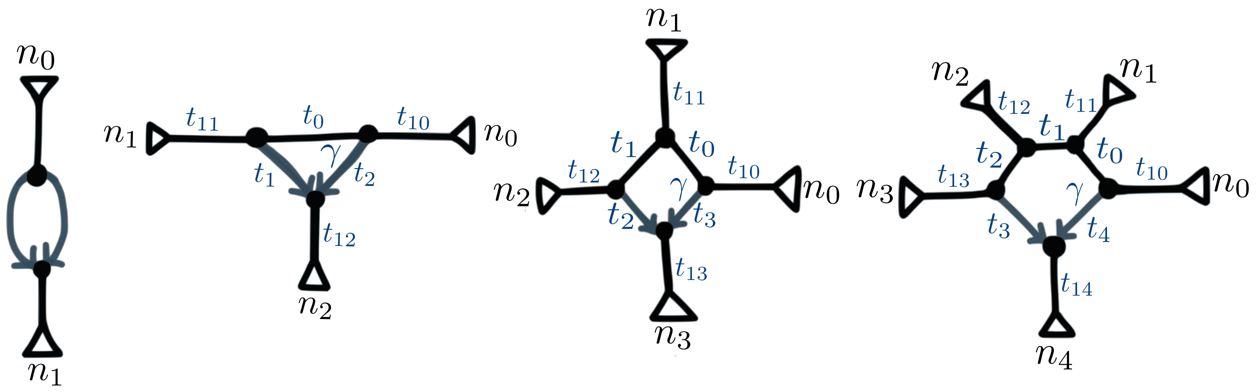

Figure S3: Characterization of n-taxon networks depending on the number of nodes in the hybridization cycle: $k = 2, 3, 4, 5$ in order from left to right.

3 Parameter identifiability

We study here whether branch lengths and heritabilities are identifiable from quartet CFs, given a fixed network topology.

3.1 Identifiability of parameters in a 5-taxon network

As mentioned before, a network with $n = 5$ taxa has 15 quartets, with quartet CFs given in section 1.1. Thus, a 5-taxon network defines 15 CF equations in the unknown parameters of branch lengths and γ. If this system has a unique solution for all the unknown parameters given a set of CF values $c_1, c_2, ..., c_{15}$, then we say that the parameters are identifiable. For example, recall the 5-taxon network in Fig. S2 (left) with system of CF equations in table S1. From a quick inspection, it is evident that not all vectors of CF values can yield a solution. For instance, the quartet CFs are identical for the 4-taxon sets ABCD and ABCE. Thus, if we consider the first 6 equations:

$$
\begin{aligned}
CF_{AB|CD} &= (1-\gamma)(1-2/3\exp(-t_1)) + \gamma(1/3\exp(-t_0)) = c_1 \\
CF_{AD|BC} &= (1-\gamma)(1/3\exp(-t_1)) + \gamma(1-2/3\exp(-t_0)) = c_2 \\
CF_{AC|BD} &= (1-\gamma)(1/3\exp(-t_1)) + \gamma(1/3)\exp(-t_0) = c_3 \\
CF_{AB|CE} &= (1-\gamma)(1-2/3\exp(-t_1)) + \gamma(1/3\exp(-t_0)) = c_4 \\
CF_{AE|BC} &= (1-\gamma)(1/3\exp(-t_1)) + \gamma(1-2/3\exp(-t_0)) = c_5 \\
CF_{AC|BE} &= (1-\gamma)(1/3\exp(-t_1)) + \gamma(1/3)\exp(-t_0) = c_6,
\end{aligned}
$$

it is obvious that a solution can exist only if

$$c_1 = c_4, \quad c_2 = c_5, \quad \text{and } c_3 = c_6.$$

In addition to these three conditions, there are many others that the CF values $c_1, c_2, ..., c_{15}$ need to fulfill for a solution to exist. In other words, the structure of the network imposes

conditions on the CF values that need to be satisfied for the system of equations to be consistent. We will call these conditions *invariants*, which are like *consistency checks* for the system. On a tree, the invariants are easy to list. For the tree in Fig. S2, for example, equating the expected quartet CFs from table S2 to a set of CF values c_i gives the following system of equations for the 2 unknown branch lengths u_1 and u_2:

$$
\begin{aligned}
CF_{AB|CD} &= 1 - 2/3\, e^{-u_1} = c_1 \\
CF_{AD|BC} &= 1/3\, e^{-u_1} = c_2 \\
CF_{AC|BD} &= 1/3\, e^{-u_1} = c_3 \\
CF_{AB|CE} &= 1 - 2/3\, e^{-u_1} = c_4 \\
CF_{AE|BC} &= 1/3\, e^{-u_1} = c_5 \\
CF_{AC|BE} &= 1/3\, e^{-u_1} = c_6 \\
CF_{AC|DE} &= 1 - 2/3\, e^{-u_2} = c_7 \\
CF_{AD|CE} &= 1/3\, e^{-u_2} = c_8 \\
CF_{AE|CD} &= 1/3\, e^{-u_2} = c_9 \\
CF_{BC|DE} &= 1 - 2/3\, e^{-u_2} = c_{10} \\
CF_{BD|CE} &= 1/3\, e^{-u_2} = c_{11} \\
CF_{BE|CD} &= 1/3\, e^{-u_2} = c_{12} \\
CF_{AB|DE} &= 1 - 2/3\, e^{-u_1-u_2} = c_{13} \\
CF_{AD|BE} &= 1/3\, e^{-u_1-u_2} = c_{14} \\
CF_{AE|BD} &= 1/3\, e^{-u_1-u_2} = c_{15}.
\end{aligned}
$$

Several equations are obviously identical. Hence, these conditions on the c_i values are necessary for the system to have a solution (u_1, u_2):

$$c_1 = c_4, \quad c_2 = c_3 = c_5 = c_6, \quad c_7 = c_{10}, \quad c_8 = c_9 = c_{11} = c_{12} \quad \text{and } c_{14} = c_{15}.$$

In addition, the three CF from each 4-taxon set must add up to 1:

$$c_1 + c_2 + c_3 = c_4 + c_5 + c_6 = c_7 + c_8 + c_9 = c_{10} + c_{11} + c_{12} = c_{13} + c_{14} + c_{15} = 1.$$

Finally, three times the minor CF of ABCD multiplied by three times the minor CF of ACDE should be equal to three times the minor CF of ABDE. This is because $\exp(-u_1)\exp(-u_2)$ should be equal to $\exp(-u_1 - u_2)$. We thus obtain the following additional invariant

$$(3c_2)(3c_8) = 3c_{14}.$$

For this species tree, we obtained 13 *independent* invariants that the c_i values must satisfy for the system to have a solution. If the values $c_1, c_2, ..., c_{15}$ are obtained from a tree with branch lengths u_1^*, u_2^* (using table S2), then these c_i values automatically satisfy the invariants of the system, and $(u_1, u_2) = (u_1^*, u_2^*)$ is necessarily one solution of the system of equations. Our hope is that this solution is unique.

The question of identifiability can now be restated in terms of the number of algebraically independent equations that the system has. For the tree above, there are 15 original equations but 13 independent invariants, therefore two algebraically *independent* equations. Since we only have 2 unknown parameters u_1 and u_2, we can solve for them. We can do a similar analysis with the network example in Fig. S2 with CF equations in table S1. We have a system of 15 equations, and we would like to know how many independent invariants are defined by this system in order to determine how many algebraically independent equations we have. We know from algebraic geometry [2] that a system with the same number of algebraically independent equations as unknown parameters has finitely many solutions. This does not imply that the parameters are identifiable, but it does imply that the parameters are generically indentifiable. It also implies that, given perfect data (infinitly many genes, correctly reconstructed gene trees), the pseudolikelihood has finitely many maxima. Proving uniqueness of solution is a hard problem in algebraic geometry and is beyond the scope of the present work.

Therefore, to study the generic identifiability of parameters, we obtained the system of quartet CF equations for each network, and we verified whether the number of algebraically independent equations was equal to the number of parameters. We automated this using Macaulay2.

The network in Fig. S2 needs four parameters $(\gamma, t_0, t_1, t_{11})$ and 15 equations. In this example, the 15 equations have 12 independent invariants. Thus, we only have three algebraically independent equations and four parameters. This means that the system has an infinite number of solutions and we cannot solve for γ, t_0, t_1, t_{11}.

In this case, which is a bad diamond I, we decided to reparametrize $(\gamma, t_0, t_1, t_{11})$. The quartet CFs can be expressed in terms of the following 3 parameters only, which are identifiable from the 3 algebraically independent equations: $x = \gamma(1-\exp(-t_0))$, $y = (1-\gamma)(1-\exp(-t_1))$ and t_{11}.

They have the following interesting interpretation: the lineage from species B in Fig. S2 (left) either originated from the hybridization edge and coalesced with C along the edge of length t_0 with probability $x = \gamma(1-\exp(-t_0))$, or it originated from the other hybridization edge and coalesced with A along the edge of length t_1, with probability $y = (1-\gamma)(1-\exp(-t_1))$.

3.2 *n*-taxon network: parameter identifiability when $h = 1$

In this section we assume that the network has a single hybridization (assumption relaxed in the next section) and we seek to determine if the parameters around the cycle created by this hybridization are identifiable (Fig. S3), given the network topology. Parameter identifiability depends on the number of nodes in the cycle created by the hybridization event. We summarize the results below.

For $\boldsymbol{k = 3}$, the parameters are not identifiable if $n \leq 5$. If $n \geq 6$ and $n_i \geq 2$ for all $i = 0, 1, 2$ (see Fig. S3), we have 6 algebraically independent equations and 7 parameters. Thus, the 7 parameters are not identifiable, but 6 of them are identifiable if the remaining

parameter if known. We decided to set $t_{12} = 0$ and estimate the other 6 paramerers. We call this case a *good triangle*.

For $\boldsymbol{k = 4}$, all parameters are identifiable if either $n_0 \geq 2$ (or n_2, symmetrically), or if both n_1 and $n_3 \geq 2$ (see Fig. S3). Parameters are not all identifiable in the remaining 2 cases, which we call *bad diamonds* I and II (see Fig. S3). The bad diamond I was described in the previous section. For a bad diamond II, 6 parameters are needed around the hybridization and we found only 5 algebraically independent equations, with no simple reparametrization. So, we set $t_{13} = 0$ and solve for the remaining parameters.

For $\boldsymbol{k = 5}$ all parameters are identifiable. To show it, we first considered the case when $n_0 = \cdots = n_4 = 1$, that is, we considered information from quartets with at most one taxon from each subnetwork. Using Macaulay2 we found that γ and the 3 tree edge lengths in the cycle (t_0, t_1, t_2) were identifiable. Next, we used previous results from $k = 4$. If $n_i \geq 2$ and $i \neq 4$, the length t_{1i} of the edge attaching subnetwork i is identifiable because we can extract a good diamond from which we can identify t_{1i}. If $n_4 \geq 2$, the hybrid branch lengths t_3, t_4 and the subnetwork branch length t_{14} are needed. To identify these parameters, we can extract a bad diamond II, which originally provided 6 algebraically independent equations. These are enough to identify the remaining 3 unknown branch lengths.

For $\boldsymbol{k > 5}$ we can prove that all needed parameters are identifiable by extracting subnetworks with $k = 5$, each identifying a different subset parameters, together spanning all parameters.

Note that the branch lengths labelled in Fig. S3 are not all needed for all networks. In particular, if $n_i = 1$ then the branch leading to subnetwork i is an external branch, and its length t_{1i} is irrelevant for any CF. For a bad diamond I for example ($n_1 \geq 2$ but other $n_i = 1$), t_{10}, t_{12} and t_{13} are irrelevant and obviously non-identifiable. In this bad diamond I, the hybrid branch lengths t_2 and t_3 are also irrelevant, as they only have 1 descendant ($n_3 = 1$) like external edges. These branch lengths were omitted from our study of identifiability: we did not include them in the list of parameters to be studied and we excluded them from the list of parameters during the pseudolikelihood optimization search.

3.3 Parameter identifiability in level-1 networks

We now extend the results from the previous section to networks with $h > 1$, provided that the network is of level 1. As already noted in section 2.2, only a subset of quartets bear information on the hybridization of interest and on parameters around this hybridization: those involving $n_i \leq 2$ taxa from any given subnetwork i. Because the network is of level 1, any hybridization in subnetwork i does not affect the quartet CFs if $n_i = 0$ or $n_i = 1$. For 4-taxon sets that need to involve $n_i = 2$ taxa from subnetwork i, we noted in section 2.2 that the subnetwork reduced to these 2 taxa was equivalent (in terms of quartet CFs) to a subnetwork with no hybridizations where the 2 taxa are sister (subnetwork of type 1 in section 1.2). This may come at the cost of having to transform the length t_{1i} of the branch linking the cycle from the hybridization of interest to subnetwork i (Fig. S3). If there is at least one pair of taxa from subnetwork i such that the equivalent subnetwork reduced to this

pair is separated from the cycle by a branch of untransformed length t_{1i}, then the results stated in the previous section apply directly. If there is no such pair of taxa from subnetwork i, then the results stated in the previous section apply to the transformed branch length $\tilde{t}_{1i}$ instead.

4 Heuristic search in the space of networks

The search is initialized with a user-specified network, which could be a tree obtained with a very fast quartet-based tree estimation method like ASTRAL [3]. If a tree topology is given with no branch lengths, those are initialized using the average observed CF of the quartets that span that branch exactly, $\overline{\text{CF}}$, transformed to coalescent units by $t = -\log(1 - 3/2\,\overline{\text{CF}})$. By default, SNaQ performs 10 independent searches, each using a starting topology based on the user-specified network or tree. For each search, an NNI is performed on the user-specified topology with probability 0.7 by default, so that independent runs have different starting topologies. If the starting topology is a network, then with probability 0.7 by default the origin (or target) of a hybrid edge is moved. Each search then navigates the network space by altering the current network using one of 5 proposals, chosen at random:

1. Move the origin of an existing hybrid edge. A hybrid node is chosen uniformly at random, then one of the 2 parent edges of that node is chosen according to the inheritance probabilities: the edge with inheritance γ is chosen with probability $1-\gamma$. A new edge is then chosen at random from the vicinity of the current origin. This edge is cut into 2 smaller edges to insert the new origin. The two branches at the old origin are merged along with their branch lengths, whose proportions are used for the 2 new branches created around the new origin.

2. Move the target of an existing hybrid edge, similarly to the previous move.

3. Change the direction of an existing hybrid edge. A hybrid node is chosen uniformly at random, then one of the 2 parent edges of that node is chosen according to the inheritance probabilities: the edge with inheritance γ is chosen with probability $1-\gamma$. The direction of the chosen hybrid edge is flipped. The former hybrid node becomes a tree node and the other node attached to the hybrid edge becomes a hybrid node. Branch lengths are left unchanged.

4. Perform a nearest-neighbor interchange (NNI) on a tree edge, around a tree edge chosen uniformly at random. [4] describe a similar type of NNI that yields a level-1 (unrooted) network, and showed that the resulting network is level-1 if the chosen tree edge is not a link. Branch lengths are left unchanged.

5. Add a hybridization if the current topology has $h < h_m$. Two tree edges are chosen at random and a new hybrid edge is created between these 2 edges. The new nodes (origin and target) are placed uniformly along each chosen edge. γ is drawn uniformly in $(0, 0.5)$. The new edge length is initialized at 0. If the new cycle intersects a

previously existing cycle, the origin or the target is chosen at random, to be moved to a neighboring edge immediately.

Any new proposed network is checked to verify that it is of level 1, with $h \leq h_m$ and with at least one valid placement for the root. If not, the move fails immediately and a new move is proposed at random. The search continues until one the following criterion is reached:

- the absolute difference between the pseudolikelihood value of the newly accepted and the current network is smaller than a tolerance threshold, 0.001 by default.
- the pseudo-deviance (difference between the theoretical maximum pseudolikelihood and the network pseudolikelihood) is below the tolerance threshold.
- the number of failed moves reaches a limit, 100 by default.
- for all move types, the number of failed attempts reaches a limit, which is specific to the proposal type, n and h. This is to avoid repeated proposals of the same new network. For example, on a network with $h = 1$, there are at most $N_1 = 8$ ways to move the origin of either hybrid edge to a neighboring location. For this network, we chose a limit of 28 failed moves of type 1 to ensure that all of the 8 distinct proposals were attempted with high probability, based on the theory of the coupon's collector [5]. In general, for $N = N_i$ distinct ways to attempt a move of type i, the upper threshold was set to $N\sum_{i=1}^{N}\frac{1}{i}+\sqrt{\frac{\pi}{6}}N$. We calculated N_i for each move type. In general there are $N_1 = N_2 = 8h$ ways to move a hybrid origin or target and $N_3 = 2h$ ways to change a hybrid edge direction. There are $2n - 3$ internal tree edges on n taxa, so there are $N_4 = 2n - 3$ ways to propose an NNI and $N_5 = \binom{2n-3}{2}$ ways to choose two tree edges to add a new hybridization.

5 Identifiability from quartets versus triples

A pseudolikelihood based on rooted triples is used in [6] to estimate a rooted network, whereas we use unrooted quartets to estimate a semi-directed network. We show here that unrooted quartets provide more information to identify networks, that may not be identifiable with rooted triples. For example, [6] present two networks Ψ_1 and Ψ_2 that are not identifiable by their set of triples (see Fig. S4). We show here that these networks are identifiable from the set of quartets.

A rooted triple is equivalent to an unrooted quartet by adding the outgroup used to root the triple, if gene trees were initially rooted using an outgroup. If gene trees were rooted using a molecular clock assumption or midpoint rooting, for example, the root still implies an outgroup shared by all gene trees. Thus, the full set of rooted triples correspond to the unrooted quartets on any 3 ingroup taxa and the outgroup (see table S3). With 4 ingroup taxa, the collection of 4-taxon subsets has one extra set that provides enough information

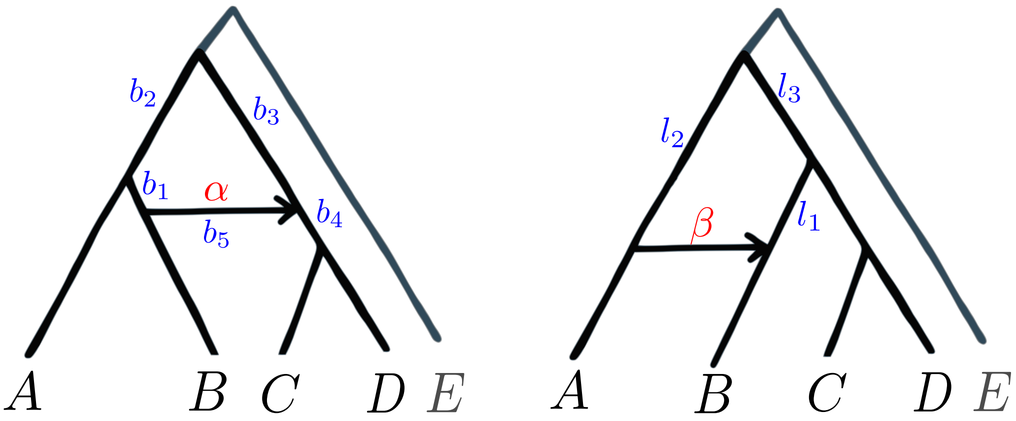

(a) Ψ_1 (b) Ψ_2

Figure S4: Example from [6] of networks non-identifiable with rooted triples from the 4 ingroup taxa (A, B, C, D). These two networks are identifiable from each other based on unrooted quartets from all 5 taxa.

Rooted triples	ABC	ACD	ABD	BCD	
Unrooted quartets	ABCE	ACDE	ABDE	BCDE	ABCD

Table S3: The collection of 3-taxon subsets from a 4-taxon rooted network corresponds in part to the collection of 4-taxon subsets from an unrooted network on the same 4 taxa (A,B,C,D) plus an outgroup (E). Extra subsets are obtained by sampling ingroup taxa only, like ABCD.

to distinguish between the two networks in Fig. S4. Fig. S4 corresponds to the networks Ψ_1 and Ψ_2 in [6] with an added outgroup E. As mentioned in previous sections, the 15 CF equations are not independent as they need to add up to one for any given 4-taxon subset (or 3-taxon subset) and some subsets are of type 1 so the two minor CFs are equal. The independent formulas for the CFs from Ψ_1 with added outgroup E are:

$$
\begin{aligned}
CF_{BC|AE} &= (1-\beta)(1-2/3\,e^{-l_3}) + \beta 1/3\,e^{-l_2} \\
CF_{BA|CE} &= (1-\beta)1/3\,e^{-l_3} + \beta(1-2/3\,e^{-l_2}) \\
CF_{BE|CD} &= (1-\beta)(1-2/3\,e^{-l_1}) + \beta(1-2/3\,e^{-l_3-l_1}) \\
CF_{AE|CD} &= 1-2/3\,e^{-l_1-l_3} \\
CF_{AB|CD} &= (1-\beta)(1-2/3\,e^{-l_1}) + \beta(1-2/3\,e^{-l_3-l_2-l_1})
\end{aligned}
$$

whereas the formulas from Ψ_2 are

$$
\begin{aligned}
CF_{BC|AE} &= \alpha(1 - 2/3\, e^{-b_1}) + (1-\alpha)1/3\, e^{-b_2} \\
CF_{BA|CE} &= \alpha 1/3\, e^{-b_1} + (1-\alpha)(1 - 2/3\, e^{-b_2}) \\
CF_{BE|CD} &= \alpha^2(1 - 2/3\, e^{-b_4-b_5}) + 2\alpha(1-\alpha)(1 - e^{-b_4} + 1/3\, e^{-b_2-b_4-b_1}) + (1-\alpha)^2(1 - 2/3\, e^{-b_4-b_3}) \\
CF_{AE|CD} &= \alpha^2(1 - 2/3\, e^{-b_4-b_5-b_1}) + 2\alpha(1-\alpha)(1 - e^{-b_4} + 1/3\, e^{-b_2-b_4}) + (1-\alpha)^2(1 - 2/3\, e^{-b_4-b_3}) \\
CF_{AB|CD} &= \alpha^2(1 - 2/3\, e^{-b_4-b_5}) + 2\alpha(1-\alpha)(1 - e^{-b_4} + 1/3\, e^{-b_4-b_1}) + (1-\alpha)^2(1 - 2/3\, e^{-b_4-b_3-b_2}).
\end{aligned}
$$

If we set $b_2 = 1, b_3 = 2, b_4 = 1, b_5 = 0, b_1 = 1, \alpha = 0.1, \beta = 0.663163, l_2 = 1.951019, l_3 = 0.207841, l_1 = 1.841435$ as in [6], we see that the first 4 CFs are identical between Ψ_1 and Ψ_2, but the fifth one (corresponding to the subset without the outgroup, not present in the triples) differs between Ψ_1 and Ψ_2, allowing us to distinguish between both networks.

$$
\begin{aligned}
CF_{BC|AE}(\Psi_1) &= CF_{BC|AE}(\Psi_2) = 0.18584 \\
CF_{BA|CE}(\Psi_1) &= CF_{BA|CE}(\Psi_2) = 0.69153 \\
CF_{BE|CD}(\Psi_1) &= CF_{BE|CD}(\Psi_2) = 0.90743 \\
CF_{AE|CD}(\Psi_1) &= CF_{AE|CD}(\Psi_2) = 0.914114 \\
CF_{AB|CD}(\Psi_1) = 0.92956 &\neq CF_{AB|CD}(\Psi_2) = 0.956292.
\end{aligned}
$$

6 Simulated data

Gene trees were simulated on the networks shown below.

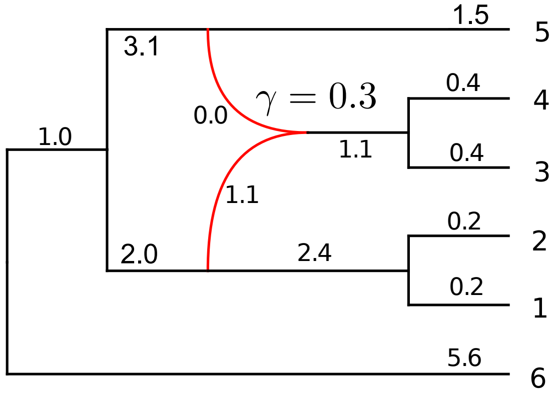

(a) $n = 6$ taxa, $h = 1$ hybridization ($k = 4$)

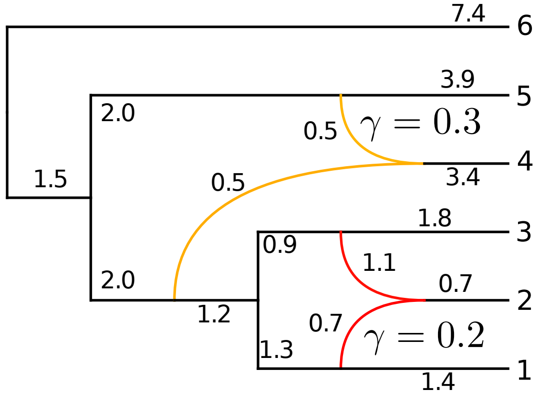

(b) $n = 6$ taxa, $h = 2$ hybridizations ($k = 4, 4$)

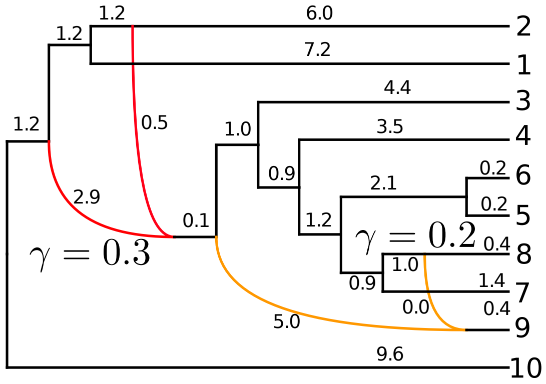

(c) $n = 10$ taxa, $h = 2$ hybridizations ($k = 4, 7$)

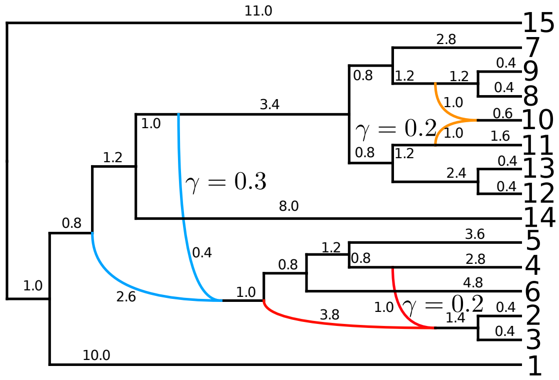

(d) $n = 15$ taxa, $h = 3$ hybridizations ($k = 4, 5, 6$)

Figure S5: True networks used for the simulations, including a bad diamond I (top right, $n = 6$, with $\gamma = 0.2$) and a bad diamond II (bottom left, $n = 10$, with $\gamma = 0.3$). Branch lengths are in coalescent units.

7 *Xiphophorus* fish network analysis

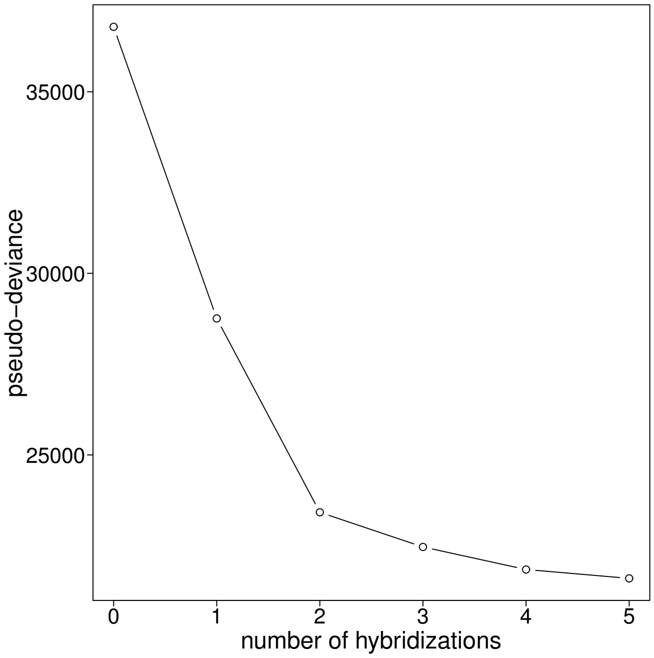

Figure S6: Pseudo-deviance score vs number of hybridizations for the *Xiphophorus* fish data

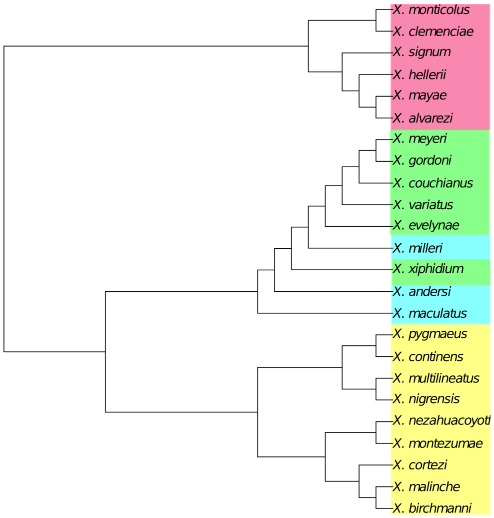

(a) $h = 0$

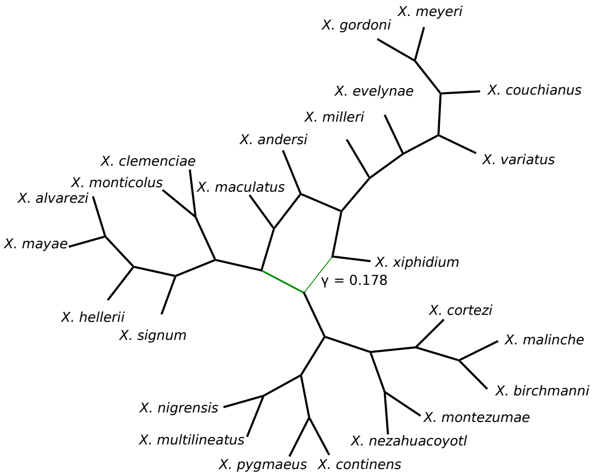

(b) $h = 1$

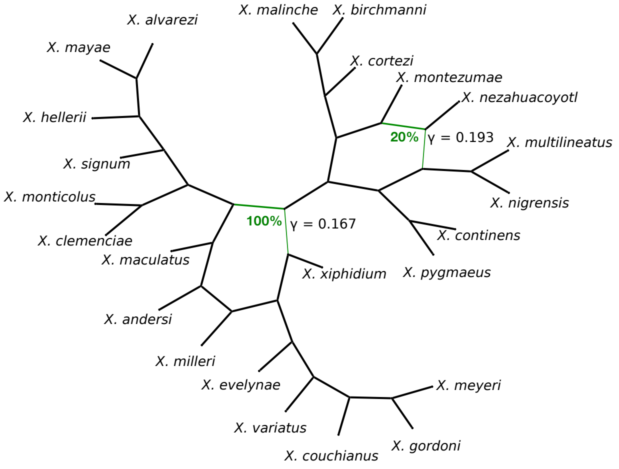

(c) $h = 2$

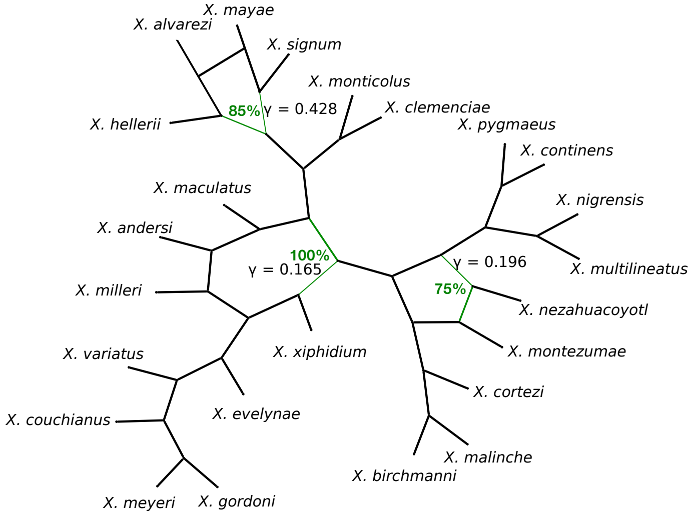

(d) $h = 3$

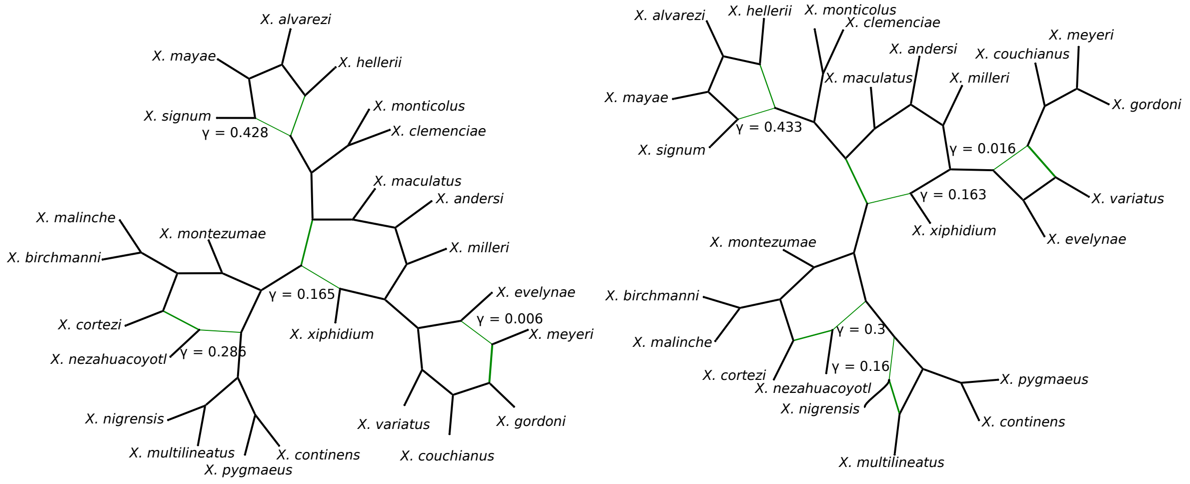

(e) $h = 4$ (f) $h = 5$

Figure S7: Estimated networks for the *Xiphophorus* fish data for $h = 0$ to 5. The estimated tree ($h = 0$) is rooted with the southern swordtail (SS) outgroup clade (pink). Networks with $h \geq 1$ are shown as semi-directed networks, as estimated. Hybrid edges, shown in green, are directed toward their hybrid node, which is the node where the two hybrid edges meet and represents an ancestral species of mixed parental origins. The minor hybrid edge is thinner and annotated with the inheritance probability (γ) whereas the major hybrid edge is thicker and its inheritance probability is given by $1 - \gamma$ (not shown). With $h \geq 3$, the direction of one hybridization in the SS clade conflicts with placing the root at the base of this clade (see main text). In the estimated network with $h = 2$ and 3, numbers in green represent bootstrap support for a given hybridization. In particular, the 75% for $h = 3$ (or 20% for $h = 2$) support value refers to the full hybridization cycle, including the placement of *X. nezahuacoyotl* as sister to *X. montezumae* in the major tree. *X. nezahuacoyotl* was placed differently (sister to the clade *X. cortezi+X. birchmanni+X. malinche*) in 3 bootstrap networks, for which the inferred reticulation still had *X. nezahuacoyotl* as the recipient lineage. It also had the same donor lineage as shown in (c) or in (d).

References

[1] Huson D, Rupp R, Scornavacca C. 2010. Phylogenetic Networks. New York, NY: Cambridge University Press, 1st editio edition.

[2] Cox D, Little J, O'Shea D. 2007. Ideals, varieties, and algorithms. Springer, third edition.

[3] Mirarab S, Reaz R, Bayzid MS, Zimmermann T, Swenson MS, Warnow T. 2014. ASTRAL: genome-scale coalescent-based species tree estimation. *Bioinformatics.* 30:i541–i548.

[4] Huber KT, Linz S, Moulton V, Wu T. 2015. Spaces of phylogenetic networks from generalized nearest-neighbor interchange operations. *Journal of Mathematical Biology.* In press.

[5] Feller W. 1950. Introduction to Probability Theory vol. I. New York, NY: Wiley, third edition.

[6] Yu Y., Nakhleh, L. A maximum pseudo-likelihood approach for phylogenetic networks. *BMC Genomics 2015*, 16(Suppl 10): S10.

[7] Cui R, Schumer M, Kruesi K, Walter R, Andolfatto P, Rosenthal GG. 2013. Phylogenomics reveals extensive reticulate evolution in *Xiphophorus* fishes. *Evolution; international journal of organic evolution.* 67:2166–79.
